# Genome Identification of B-BOX Gene Family Members in Seven *Rosaceae* Species and Their Expression Analysis in Response to Flower Induction in *Malus domestica*

**DOI:** 10.3390/molecules23071763

**Published:** 2018-07-18

**Authors:** Abdullah Shalmani, Sheng Fan, Peng Jia, Guofang Li, Izhar Muhammad, Youmei Li, Rahat Sharif, Feng Dong, Xiya Zuo, Ke Li, Kun-Ming Chen, Mingyu Han

**Affiliations:** 1 College of Horticulture, Northwest A&F University, Yangling 712100, China; abdullqadir36@yahoo.com (A.S.); likessd@126.com (S.F.); jiapeng1034@nwsuaf.edu.cn (P.J.); guofangli@nwsuaf.edu.cn (G.L.); lym890525@163.com (Y.L.); rahatsharif2016@nwafu.edu.cn (R.S.); dong-feng@nwafu.edu.cn (F.D.); xiyazuo@163.com (X.Z.); keli505@nwafu.edu.cn (K.L.); 2 State Key Laboratory of Crop Stress Biology in Arid Areas, College of Life Sciences, Northwest A&F University, Yangling 712100, China; izeyaar@gmail.com

**Keywords:** *Rosaceae*, *BBX*, synteny, expression analysis, flower induction

## Abstract

BBX proteins play important roles in regulating plant growth and development including photomorphogenesis, photoperiodic regulation of flowering, and responses to biotic and abiotic stresses. At present, the genomes of seven *Rosaceae* fruit species have been fully sequenced. However, little is known about the *BBX* gene family and their evolutionary history in these *Rosaceae* species. Therefore, in this study total, 212 *BBX* genes were investigated from seven *Rosaceae* species (67 from *Malus* × *domestica*, 40 from *Pyrus* *communis*, 22 from *Rosa Chinesis*, 20 from *Prunus* *persica*, 21 from *Fragaria* *vesca*, 22 from *Prunus* *avium*, and 20 from *Rubus* *occidentalis*). The chemical properties, gene structures, and evolutionary relationships of the *BBX* genes were also studied. All the *BBX* genes were grouped into six subfamilies on the basis of their phylogenetic relationships and structural features. Analysis of gene structure, segmental and tandem duplication, gene phylogeny, and tissue-specific expression with the ArrayExpress database showed their diversification in function, quantity, and structure. The expression profiles of 19 *MdBBX* genes in different tissues were evaluated through qRT-PCR. These genes showed distinct transcription level among the tested tissues (bud, flower, fruit, stem, and leaf). Moreover, expression patterns of 19 *MdBBX* genes were examined during flowering induction time under flowering-related hormones and treatments (GA3, 6-BA, and sucrose). The expressions of the candidates *BBX* genes were affected and showed diverse expression profile. Furthermore, changes in response to these flowering-related hormones and treatment specifying their potential involvement in flowering induction. Based on these findings, *BBX* genes could be used as potential genetic markers for the growth and development of plants particularly in the area of functional analysis, and their involvement in flower induction in fruit plants.

## 1. Introduction

Zinc finger transcription factors are one of the most important families in the plant. They regulate different plant growth and developmental processes. Zinc finger transcription factors are classified into several subfamilies based on the structural and functional features of their individual members. Among them, B-box proteins drew more attention in the recent years because of their multiple functions. The BBX proteins are a group of zinc-finger transcription factors that contain one or two conserved domains near to N-terminus and some have additional CCT (CONSTANS, CO-like, and TIMING Of CAB1) domain near to C-terminal. The B-box domains are divided into two class, known as B-box1 (B1) and B-box2 (B2). Two B-box domains are recognized on their consensus sequence and the distance between the zinc-binding residues [1]. The segmental duplication and deletion events during evolution resulted in the differences of the consensus sequences in two B-BOX domains [2,3]. The highly conserved CCT domain is comprised of 42–43 amino acids, and it is important for the regulation of functional transcription and nuclear protein transport [4,5]. *BBXs* are important regulatory factors that control various developmental and growth process of plants including flowering, photomorphogenesis, shade avoidance, and response to biotic and abiotic stresses [6]. 

The BBX transcription factor promotes light and circadian signaling in *Arabidopsis* [1]. *CONSTANS* (known as *BBX1*) was the first investigated BBX protein in *Arabidopsis* which enhance the flowering. It regulates the transcription of the FT (Flowering Locus T) gene encoding a florigen signal, FT, which promotes flower differentiation under long-day condition [7,8]. *BBX2* and *BBX3* have less effect on flowering but overexpression of *BBX2* gene reduced the duration of two specific circadian rhythms in *Arabidopsis* [9]. Recent studies found that *AtBBX4* (COL3, CONSTANS like 3) and *AtBBX7* (*COL9*) are involved in negative regulation of CO (_CONSTANS_) and FT genes expressions, respectively [6,10,11]. *AtBBX6* promotes the expression of FT and its known as a short day-specific inducer of flowering [12]. Mutation in the *eip6* (*BBX32*) gene showed earlier flowering and enhanced the expression of flowering time and floral organ identity genes in *Arabidopsis*, while overexpression of mutant EMF1-Interacting Protein 6 (*BBX32*) display late flowering [13]. The rice CO orthologous, Hd1 (*OsBBX18*), are the *BBX* genes possessing two B-box motifs and one additional CCT domain. The function of Hd1 is parallel in the promoting of flowering time but regulates flowering during SD (short day) conditions rather than LD (long day) conditions [14]. In *Oryza sativa*, *OsCO3* containing a single B-box and CCT domain controls photoperiodic flowering [15]. The overexpression of *OsCO3* delays flowering under SD conditions in rice but does not change the flowering time in *Arabidopsis*. *OsCLO4* represses flowering under SD and LD conditions in rice [16,17]. *BBX* also plays a vital role in the regulation of flowering in other plants such as barley (*Hordeum vulgare*), beetroot (*Beta vulgaris*), chrysanthemum (*Chrysanthemum morifolium*), and grape [8,15,16,17].

BBX genes are involved in mitigating abiotic stresses. The salt tolerance protein (*STO*, *AtBBX24*) enhances the growth of roots under a highly saline condition in *Arabidopsis* [18], and was also found to trigger the salt tolerance activities in yeast cells. The salt tolerance proteins (*STO*) inoculate with CLONE EIGHTY-ONE/RADICAL-INDUCED CELL DEATH1 (CEO/RCD1) [19,20], which negatively regulates a wide range of stress-related genes [21]. *AtBBX18* act as negative regulator both in photomorphogenesis and thermotolerance in *Arabidopsis* [22]. In *Chrysanthemum*, *CmBBX24* performed a dual function, delaying flowering and also increased cold and drought tolerance in the plant [8]. Twenty-nine out of 30 rice *BBX* genes possess at least one stress-responsive *cis*-element (ARE, Wbox, GC-motif, Box-W1, HSE, and MBS), showing that these genes may express during biotic and abiotic stress [23]. Apple is a dominant fruit species cultivated in the temperate regions of the world. “Fuji” (*Malus domestica Borkh*.) is one of the most popular cultivars of apple, and it accounts for 65% planting areas in China. However, the development of the apple industry in China has enormously affected by long juvenile stage before flowering and fruiting and the poor quality of ‘Fuji’ flower buds. Furthermore, the flower initiation and flowering in many fruiting plants including apple, peach, and pears [24] occur in separate growing seasons. Thus, the study of these molecular regulatory aspects of flower induction in apple is crucial to understand and solve flowering associated complications.

Flower induction is affected by environmental and internal factors [25,26,27]. Sugar and hormones are involved in flower induction [28,29]. Among hormones, GA is the most important hormone for regulating flower induction, and the GA pathway was identified as one of the key flowering pathways [25,28,29,30]. 6-BA and sucrose promote flower bud differentiation and play a vital role in flowering induction [26,28]. In the apple genome, several flowering-related genes families have been investigated such as SPL (SQUAMOSA PROMOTER BINDING PROTEIN), IDD (INDETERMINATE DOMAIN genes), and MADS-box (DNA-binding MADS domain) [31,32]. However, little is known about the involvement of *BBX* genes and their role in flower induction in apple. Recently the apple genome has been sequenced [33] which made it possible to evaluate the structure, function, and evolution of the *BBX* gene family and their response to flower induction.

No comprehensive study of *BBX* genes in *Rosaceae* has been reported so far. Although, the *BBX* gene family has been already reported in apple and pear [34,35], we found more *BBX* homologs in apple and pear than previous works. Thus, we have performed a systematic study to analyze the *BBX* gene family and their role in flowering induction in apple. In the present study, we investigated the gene structure, phylogenetic relationships, *BBX* genes family, and their expression patterns under various flowering affecting treatments and hormones in apple. 

## 2. Results

### 2.1. The BBX Gene Family Members in Apple

**Figure 1:**
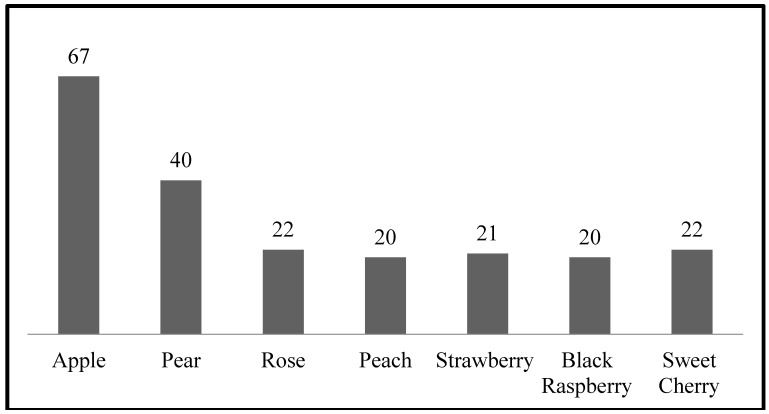
Graphical presentation of *BBX* gene members in seven *Rosaceae* species genome.

To identify and study the *BBX* genes in the *Rosaceae* genome, keyword and BLAST searches with default parameters were performed at SGN, NCBI, and other public databases. In the present study, we identified 212 *BBX* genes after removing repeated sequences and incomplete BBX domains by manual checking (Figure 1). The chemical characterization and amino acid sequence composition of *BBX* gene family members in *Rosaceae* species were predicted using the ExPaSy tool. We revealed detailed information including gene name, chromosome location, length of coding sequences and proteins, instability index, major amino acid^s,^ isoelectric point, and the molecular weight of every gene (Tables S1 and S2).

All these BBX genes have diverse length and molecular weights, ranging from 7.74 (lowest) (*MdBBX25* and *MdBBX65*) to 126.05 (highest) (*FvBBX11)* amino acid residues. Therefore varied isoelectric points and molecular weights were observed among them. The BBX genes encode proteins with the coding sequence of 189–3483 bp. The lowest coding sequence was investigated for *MdBBX25* and *MdBBX65* (189 bp), followed by *MdBBX31* (192 bp), FvBBX17 (210), *MdBBX8* (213 bp), and *MdBBX66* (237 bp) while the highest number of coding sequences were observed for *FvBBX11* (3483bp), 2nd, 3rd, and 4th positions were held by *PcBBX36* (3273bp), *PcBBX9* (2784 bp), and *MdBBX37* (2772 bp), respectively. The lowest isoelectric point (3.33) was observed for *MdBBX57*, while *MdBBX58* showed the highest value of the isoelectric point (9.91). The high number of *Rosaceae* BBX proteins members is acidic in nature according to the isoelectric point, which is lower than seven. However, the isoelectric point of some BBX protein members (*MdBBX2*, *MdBBX6*, *MdBBX12*, *MdBBX16*, *MdBBX18*, *MdBBX34*, *MdBBX35*, *MdBBX40*, *MdBBX52*, *MdBBX58*, *PcBBX5*, *PcBBX26*, *RcBBX7*, *RcBBX9*, *RcBBX11*, *PaBBX12*, *PaBBX13*, *PaBBX19*, *PaBBX20*, *RoBBX14*, *RoBBX20*, *PpBBX8*, *PpBBX12*, *FvBBX16*, and *FvBBX20*) were greater than seven, indicating that they are alkaline in nature. 

The present study grouped the majority of *Rosaceae BBX* genes into unstable proteins because the instability index of most of the genes in this family was greater than 40. However, a few *BBX* genes (*MdBBX46*, *MdBBX47*, *MdBBX48*, *MdBBX53*, *PcBBX9*, *PcBBX11*, *PcBBX36*, *RcBBX9*, *RcBBX13*, *RoBBX11*, *RoBBX15*, *PpBBX9*, *FvBBX10*, and *FvBBX11*) exhibiting instability less than 40 were categorized as stable proteins. Serine (S) was the most abundant amino acid found in BBX encoded proteins, followed by Alanine (A) and Leucine (L). The majority of *Rosaceae* BBX proteins were hydrophilic, as the grand average of hydropathicity (GRAVY) value was recorded less than 0. However, some *BBX* members such as *MdBBX5*, *MdBBX66*, *PcBBX30*, *RcBBX9*, *RcBBX13*, *FvBBX17*, and *FvBBX20* had grand average of hydropathicity (GRAVY) value more than 0 which shows hydrophobic properties. The range of aliphatic indexes of these 212 *BBX* encodes proteins from 42.99 to 100.0 (Table S2).

### 2.2. Chromosomal Localization, Structure and Multiple Sequence Alignments

**Figure 2:**
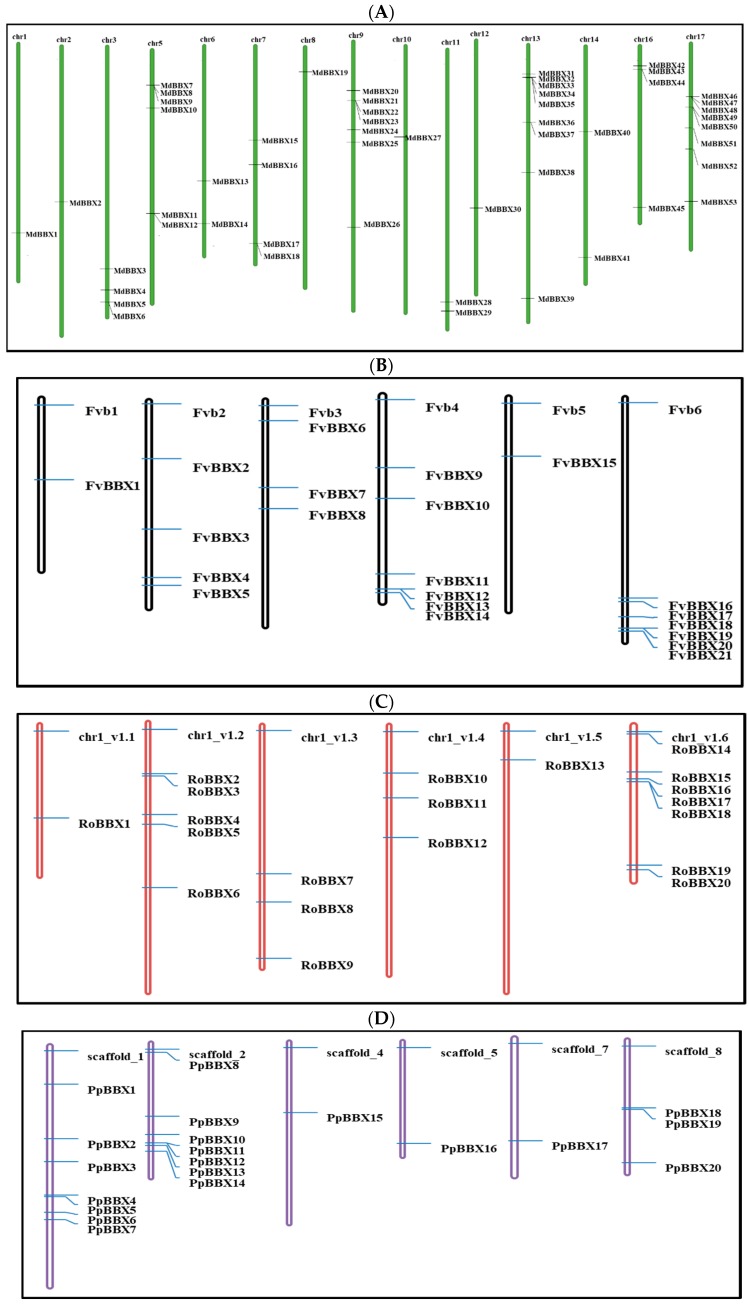

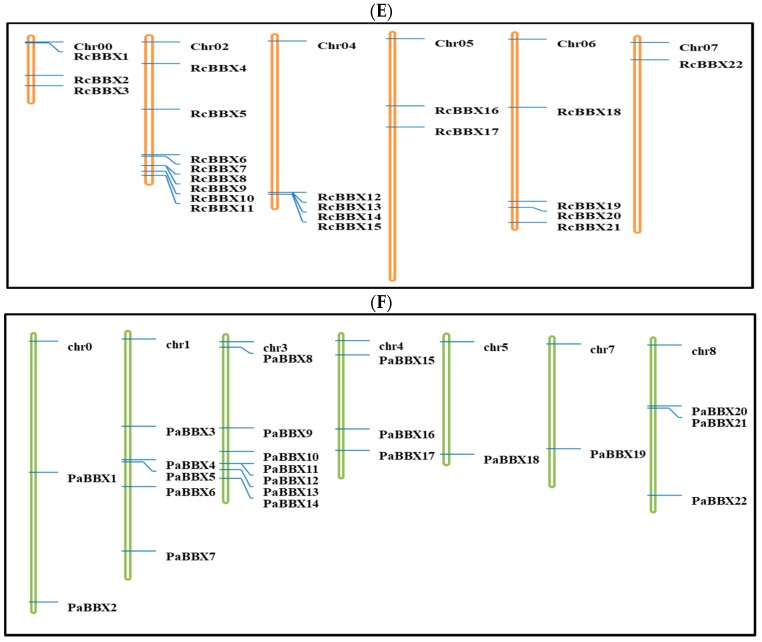
Chromosomal location of *BBX* genes on apple chromosomes. (**A**) *Malus* × *domestica*; (**B**) *Fragaria vesca*; (**C**) *Rubus occidentalis*; (**D**) *Prunus persica*; (**E**) *Rosa Chinensis*; (**F**) *Prunus avium*, respectively. The graphical view was drawn from each gene ID and scaffolds information and position of each gene are indicated by a line.

We classified the apple (*Malus domestica)*, peach (*Prunus persica*), black raspberry (*Rubus occidentalis*) strawberry (*Fragaria vesca*), sweet cherry (*Prunus avium*), and Rose (*Rosa chinensis*) *BBX* genes on the basis of their location in apple chromosomes for better understanding and consistency. The apple *BBX* genes were distributed on all chromosomes, except for chromosomes 4 and 15 (Figure 2). The highest number of *BBX* genes was found on chromosome 13 (9 *MdBBX* genes). Chromosomes 17 contained 8 *MdBBX* genes, 7 genes were found on chromosome 9 while chromosome 5 comprised of 6 *MdBBX* genes. An equal number (4) of *MdBBX* genes each were located on chromosomes 3, 7, and 16. The remaining *MdBBX* genes were distributed as 2 genes each (*MdBBX11* and *MDBBX12*) on chromosome 6, 2 genes (*MdBBX26* and *MdBBX27*) on chromosome 11, and two genes (*MdBBX38* and *MdBBX39*) on chromosome 14. Chromosomes 1, 2, 8, 10, and 12 have only one *BBX* gene.

The *BBX* genes members were noted on all chromosomes except chromosomes 3 and 6 in the peach genome. Highest numbers of peach *BBX* genes (7) were detected on chromosomes 1 and 2. The rest of the 13 *PpBBX* genes were distributed on chromosomes 4, 5, 7, and 8. The black raspberry and strawberry genome comprised of seven haploid numbers of chromosomes, respectively. The distribution of *BBX* genes on chromosomes was similar in these two species. Chromosome 6 consisted of a maximum number of *BBX* genes, and there were seven and six *BBX* genes in each black raspberry and strawberry genome, respectively. Moreover, no *BBX* gene was found on chromosome 7 in these two fruit plants. Sweet cherry *BBX* genes were observed on seven chromosomes. Seven *BBX* members were found on chromosome 3; however, no BBX protein was found on chromosomes 2 or 6. The highest number of *BBX* members was found on chromosome 2 in the rose genome. While no *BBX* member was observed on chromosome 1 in rose.

**Figure 3:**
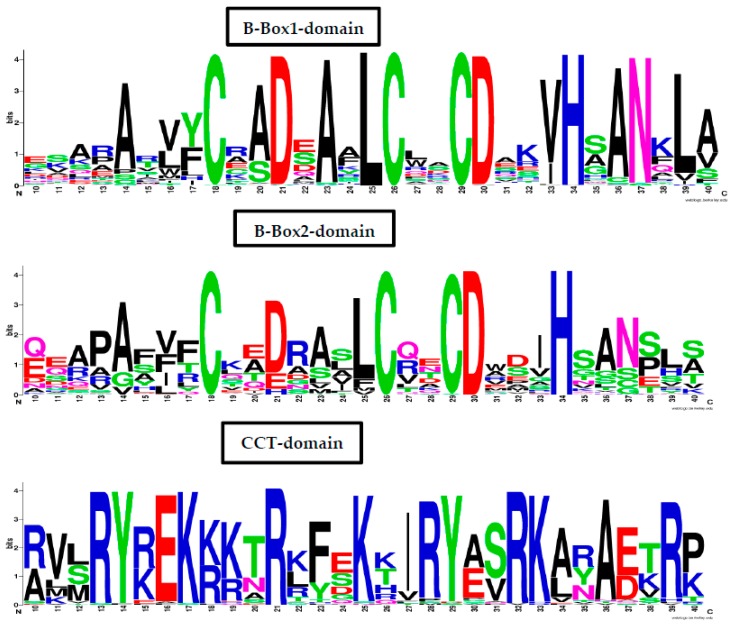
The web-logos B-Box 1, B-Box2, and CCT-domain of *Rosaceae BBX* members.

The conserved domains of the BBX proteins were confirmed by Pfam, SMART, Inter Pro Scan, Conserved Domain Database (CDD), NCBI (http://www.ncbi.nlm.nih.gov/cdd/), and Scan Prosite databases. The family-specific domains B-BOX1, B-BOX2, and CCT domain, were multiple aligned through DNAMAN software, and the logos were drawn via the Web Logo (http://weblogo.berkeley.edu/logo.cgi) online tool (Figure 3). It was previously reported that the CCT domain is the most highly conserved domain among the BBXs proteins [4,5], and a similar pattern was found in the *Rosaceae* BBX proteins (Figure S1a–c). Moreover, the B-box1 domain was found to be more conserved than B-box2 in the present study, indicating that the deletion process could occur in the B-BOX2 domain. We also found the diverse structure for all the studied proteins based on the conserved domain (Figure S2). Four different types of *BBX* genes were detected; *BBX* genes with only one B-box domain, *BBX* genes with two B-boxes domains, *BBX* genes with one B-box, and an additional CCT domain, some were found with two B-boxes and an additional CCT domain.

### 2.3. Phylogenetic and Synteny Analysis

**Figure 4:**
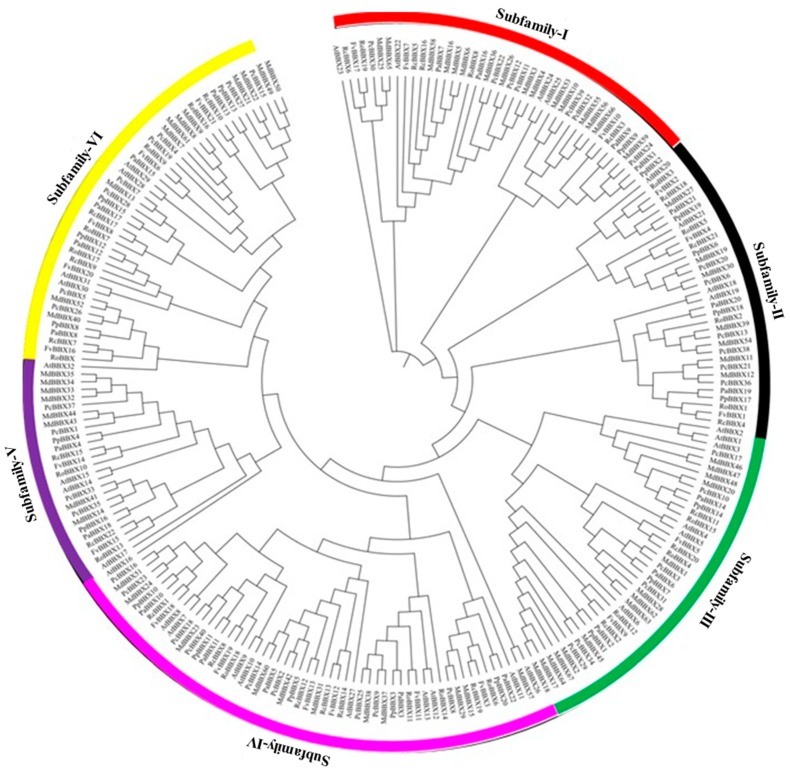
Systematic evolutionary relationships of *BBX* gene family 7 different *Rosaceae* species among six lineages within the subfamilies. The six conserved subfamilies are marked by different colors and represented as Subfamily-I, Subfamily-II, Subfamily-III, Subfamily-IV, Subfamily-V, and Subfamily-VI.

According to our analyses, the unrooted maximum-likelihood phylogenetic tree divided the *Rosaceae* BBX into 6 subfamilies (Figure 4). Most of the BBX members of subfamily I and II possessed one or two B-BOX domains. However, we found six *Rosaceae BBX* genes (*PcBBX30*, *RoBBX3*, *RoBBX5*, *PpBBX18*, *RoBBX2*, and *PpBBX19*) containing two B-BOXes and an additional CCT domain, and also four *Rosaceae BBX* gene (*PpBBX9*, *RoBBX1*, *PaBBX7*, and *RoBBX19*) owned one B-BOX and additional CCT domain in subfamily I and II. The rest of the *Rosaceae BBX* genes contained two B-BOXes and an additional CCT domain was clustered into subfamily III and IV. Additionally, we also observed the maximum numbers of *BBX* genes containing one or two B-BOXes in subfamily III and IV. One B-BOX and additional CCT domain of *Rosaceae* BBX members were identified in subfamily V and IV. A high number of one B-BOX domain containing *BBX* genes were clustered into subfamily VI along with one B-BOX and CCT containing genes. Interestingly, the present study noted quite a similar clustering for the *Arabidopsis BBX* genes (Figure 4). Two-B-BOXes domain possessing *AtBBXs* were clustered together in subfamily I and II, two B-BOXes and additional CCT domains *AtBBXs* genes were found in subfamily III and IV, *AtBBXs* having one B-BOX and CCT domain detected in subfamily V, and one B-BOX domain *AtBBXs* were identified in subfamily VI.

**Figure 5:**
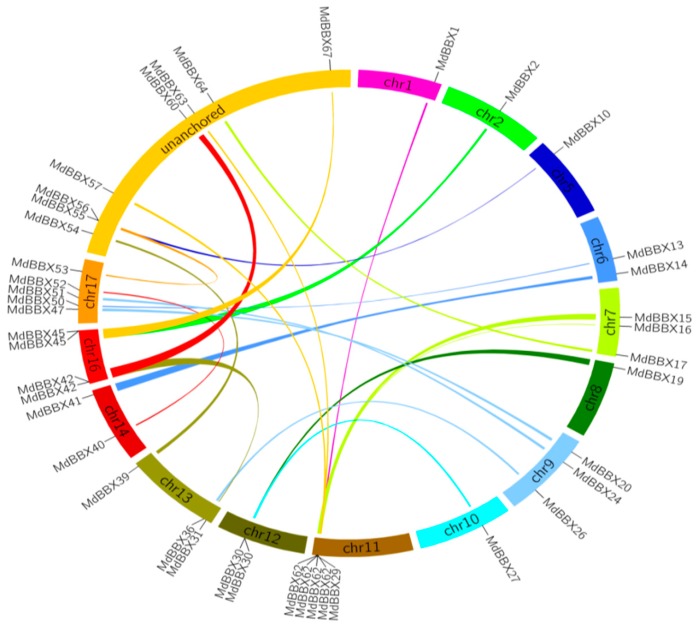
Segmental duplication of apple *BBX* genes. Genomic locations and duplicated *BBX* gene pairs in apple. Gene pairs located in the segmental duplicated chromosomal regions are linked using different lines.

**Figure 6:**
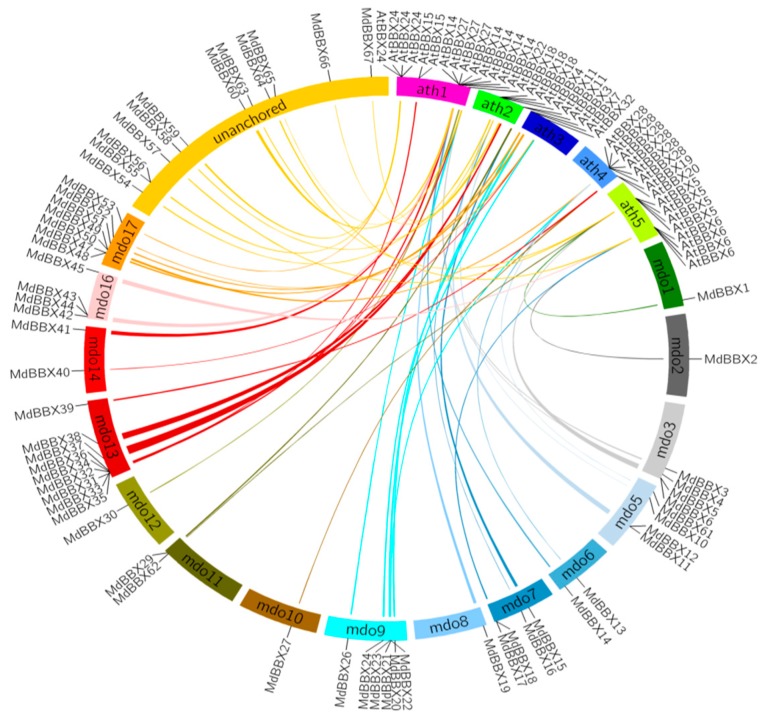
Synteny analysis of apple and *Arabidopsis BBX* genes. Chromosomes of apple and *Arabidopsis* are shown in different colors and in circular form. The approximate positions of the *AtBBX* and *MdBBX* genes are marked with a short black line on the circle. Colored curves denote the syntenic relationships between Apple and *Arabidopsis BBX* genes.

We performed the tandem duplicated analysis to study the expansion profiles of *MdBBX* genes in the apple genome (Figure 5). Segmental and tandem duplications are the main events of gene duplication. Eight pairs of *MdBBX* genes were distributed closely with each other. These genes have a similar intron-exon structure and coding length indicating that these two genes may be tandem duplicated genes. The segmentally duplicated blocks were examined within the genome to evaluate the expansion pattern of all *MdBBX* proteins. Thirty seven pairs of segmental duplicated genes were found on fourteen different apple chromosomes. However, the chromosomal location of a few genes was unknown among these thirty seven pairs of genes. We generated a comparative syntenic map based on *Arabidopsis* and apple genomes with Circos to further investigate the evolutionary relationships between *AtBBX* and *MdBBX* (Figure 6). Some ortholog genes were found between *Arabidopsis* and apple chromosomes on the basis of the syntenic map. A total of 61 *MdBBX-AtBBX* orthologous pairs were located at sixteen duplicated genomic regions between apple and *Arabidopsis*, suggesting that Apple and *Arabidopsis BBX* may have a common ancestor. Comparative genomic analysis could predict the function of apple *BBX* genes based on their *Arabidopsis* homologs.

### 2.4. Gene Structure and Motif Analysis

The conservation of gene structure in paralogous genes is sufficient to determine the evolutionary connection between introns in various circumstances. We constructed an exon-intron diagram of the *BBX* genes according to their genomic and coding sequences (Figure S3). The gene structures of all apple BBX family members were generated with GSDS software. The range of a number of introns was from one to twenty-four (*PcBBX36*) in this study. However, we found some genes without introns, only comprised of the exon. For example, *MdBBX7*, *MdBBX8*, *MdBBX9*, *MdBBX10*, *MdBBX13*, *MdBBX16*, *MdBBX25*, *MdBBX28*, *MdBBX34*, *MdBBX40*, *MdBBX52*, *MdBBX61*, *MdBBX62*, and *MdBBX65* in apple, *FvBBX6*, *FvBBX16*, and *FvBBX20* in strawberry, *RoBBX17* and *RoBBX20* gene members in black raspberry, *PcBBX5*, *PcBBX26*, *PcBBX27*, *PcBBX4*, and *PcBBX19* in pear, *RcBBX13* in rose and *PpBBX8* and *PpBBX12* in peach, respectively. The highest number of introns was observed for *PcBBX36* (24), followed by *PcBBX9* (20), *MdBBX37* (18), and *MdBBX11* (16).

The MEME tool was used to find out the motifs present in *MdBBX* members (Figure S2). MEME analysis identified a total 10 motifs in 67 *MdBBX* proteins. The Motifs were named 1–10 (Figure S3). We found that motif-2 was the largest motif based on width followed by motif-1 and motif 10. Furthermore, based on sequence analysis of motif, motif-7 was comprised more sequences compared with other nine motifs (Table 1). All the MdBBX proteins consisted of motifs 1 and 2, which suggests that all *MdBBX* proteins have a highly conserved domain. Motif 6 and 9 were present in five members (*MdBBX2*, *MdBBX45*, *MdBBX17*, *MdBBX18*, and *MdBBX64*) of the *MdBBX* family.

### 2.5. Tissue-Specific Analysis of MdBBX Expression Using ArrayExpress Data

**Figure 7:**
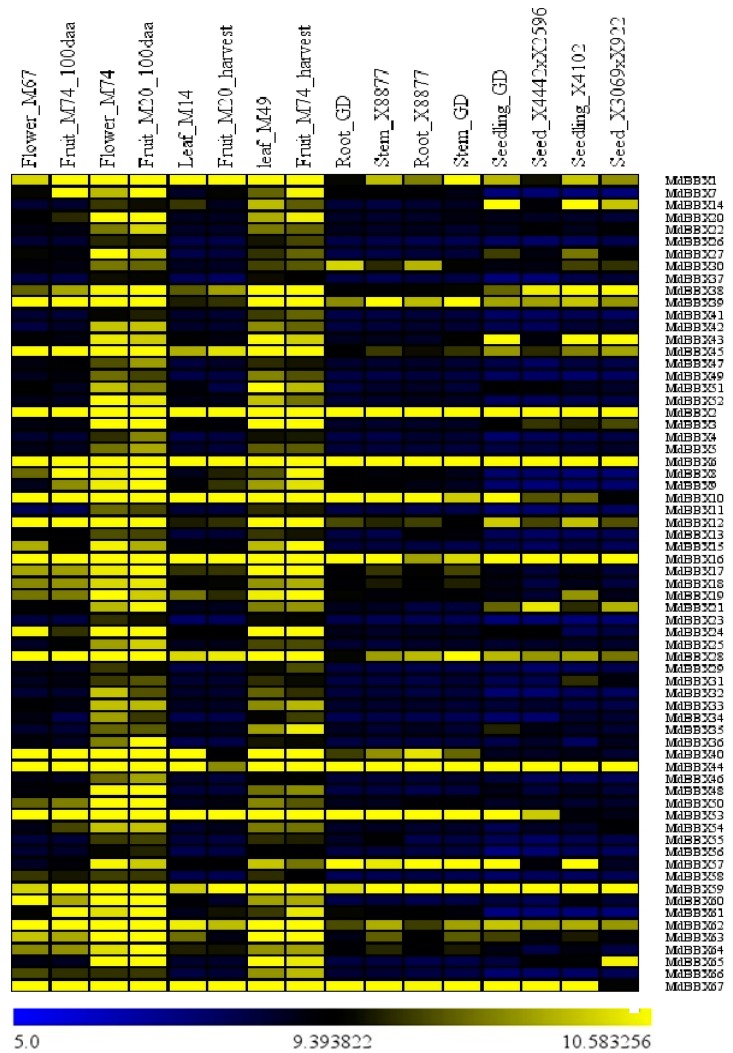
The expression profiles obtained from the ArrayExpress data, displaying diverse expression levels of apple *BBX* genes in different tissues and organs. The relative transcript level of *BBX* genes members based on ArrayExpress data was presented as heat maps from blue to yellow reflecting relative signal values; where dark blue boxes represent stronger downregulated expression and dark yellow boxes represent stronger up-regulation. 100daa referred for 100 days postanthesis, GD stand for Golden Delicious’ seedling, M74 indicating Hybrid M74, M49 indicated M49 hybrid, M20 indicated M20 hybrid, M14 indicated M14 hybrid, X8877 indicated X8877 hybrid, and X4442 and X2596 referred the cross between X4442 and X2596 varieties.

The expression pattern of genes can be useful to determine their potential role and function. Therefore, we examined the different developmental stages/tissues to study the biological roles of apple *BBX* genes in the plant growth and development, based on a set of microarray data obtained from ArrayExpress database (E-GEOD-42873) and quantitative real-time polymerase chain reaction (qRT-PCR). The expression data from the microarray analysis of apple BBX are presented in the form of a heatmap, from blue-black-yellow, reflecting the percentage expression (Figure 7). Seven tissues or organs including leaves, flowers, fruits, seeds, stems, roots, and seedlings from 10 apple varieties (M67, M74, M20, M14, M49, M74, GD, X8877, and two hybrids), were analyzed. The 67 candidates’ apple *BBX* genes displayed divergent expression pattern among the tested tissues (Figure 7). However, we found that a maximum number of apple *BBX* genes members was highly expressed in the flower_M74, fruit_M20_hurvest, leaf_M49, and fruit_M20_100daa. *MdBBX1*, *MdBBX2*, *MdBBX6*, *MdBBX10*, *MdBBX12*, *MdBBX16*, *MdBBX28*, *MdBBX39*, *MdBBX44*, *MdBBX45*, *MdBBX59*, *MdBBX62*, and *MdBBX67* were detected with a higher transcript level in all the tested tissues; however the majority number of remaining *BBX* genes showed lower expression pattern in root, seed, and seedling. The expression pattern of most of the *MdBBX* genes was similar to each other in the seedling of both varieties (seedling_GD and seedling_X4102) excluding *MdBBX19* and *MdBBX53* genes. The transcribed levels of the apple BBX members were fairly similar in stem_GD, root_X8877, stem_X8877, and root_GD. Overall, the transcript profiles of the *BBX* gene family members were high in flower, fruit, and leaf related tissues. The results demonstrated the multiple biological roles of *BBX* gene family in the plant growth and development.

### 2.6. Organ-Specific Expression of BBX Genes in Apple

**Figure 8:**
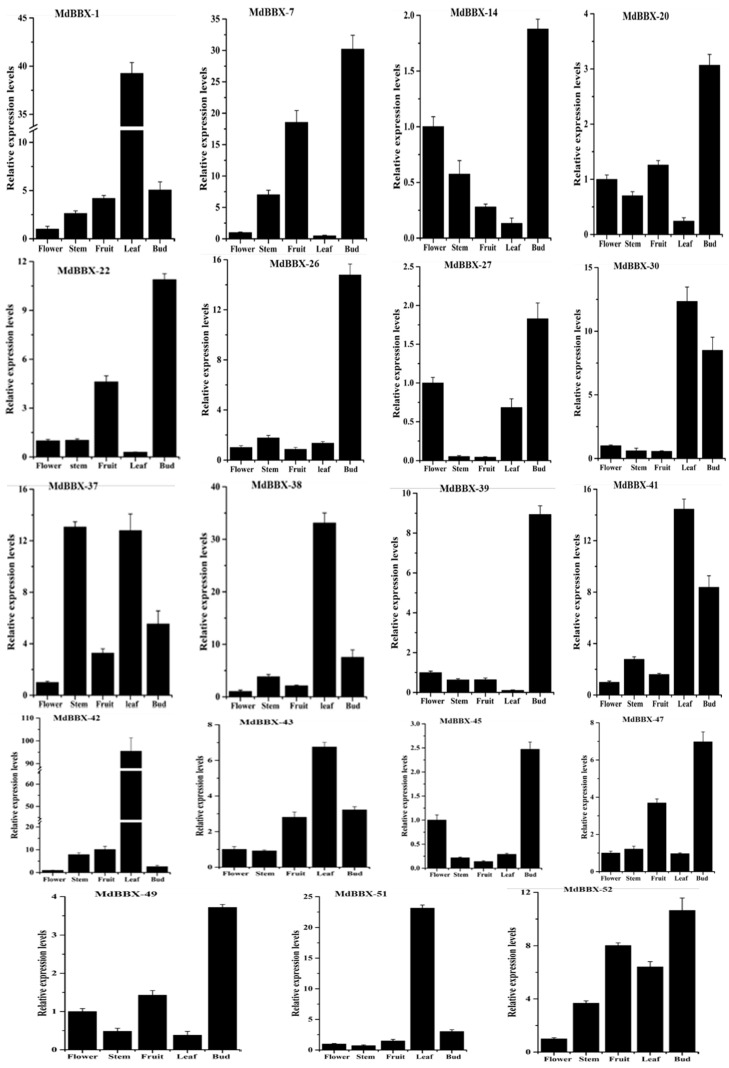
Expression profile of the *MdBBX* genes in tested tissues. The graphs indicate tissue-specific expression levels in the apple plant. The samples were collected in different developmental stages and were analyzed through qRT-PCR. The *x*-axis indicates the tissues. The *y*-axis shows the relative expression. The error bars indicate the standard deviations of the three independent qRT-PCR replicates.

Quantitative RT-PCR was carried out of the different tissues including flower, stem, fruit, leaf, and bud to evaluate the expression pattern of 19 *MdBBX* genes (Figure 8). The 19 *MdBBX* genes were selected based on our previous transcription data [26]. The actin gene served as an endogenous control. We found that all the studied genes of apple BBX family showed high expression profiles in bud tissue compared with other four tested tissues except *MdBBX1*, *MdBBX30*, *MdBBX37*, *MdBBX38*, *MdBBX41*, *MdBBX42*, *MdBBX43*, and *MdBBX51* (Figure 8). We also observed considerable expression for *MdBBX1*, *MdBBX30*, *MdBBX37*, *MdBBX38*, *MdBBX41*, *MdBBX42*, *MdBBX43*, and *MdBBX51* genes in bud but lower level than flower, fruit, stem and leaf tissues. In leaf, *MdBBX1*, *MdBBX30*, *MdBBX37*, *MdBBX38*, *MdBBX41*, *MdBBX42*, *MdBBX43*, *MdBBX51*, and *MdBBX52* exhibited high transcribed level. The transcript levels of the studied apple BBX members were low in flower tissue. Some genes such as *MdBBX7*, *MdBBX20*, *MdBBX22*, *MdBBX37*, *MdBBX43*, *MdBBX47*, and *MdBBX52* were detected with moderate transcript level in fruit. The expressions of apple BBX members were less in stem except for *MdBBX7*, *MdBBX37*, *MdBBX41*, and *MdBBX42*, showed reasonable expression pattern. The results further strengthen the evidence came from the microarray data that *BBX* gene family control various aspects of the plant during growth and development.

### 2.7. Expression of BBX Genes in Response to Flowering Affecting Treatments

**Figure 9:**
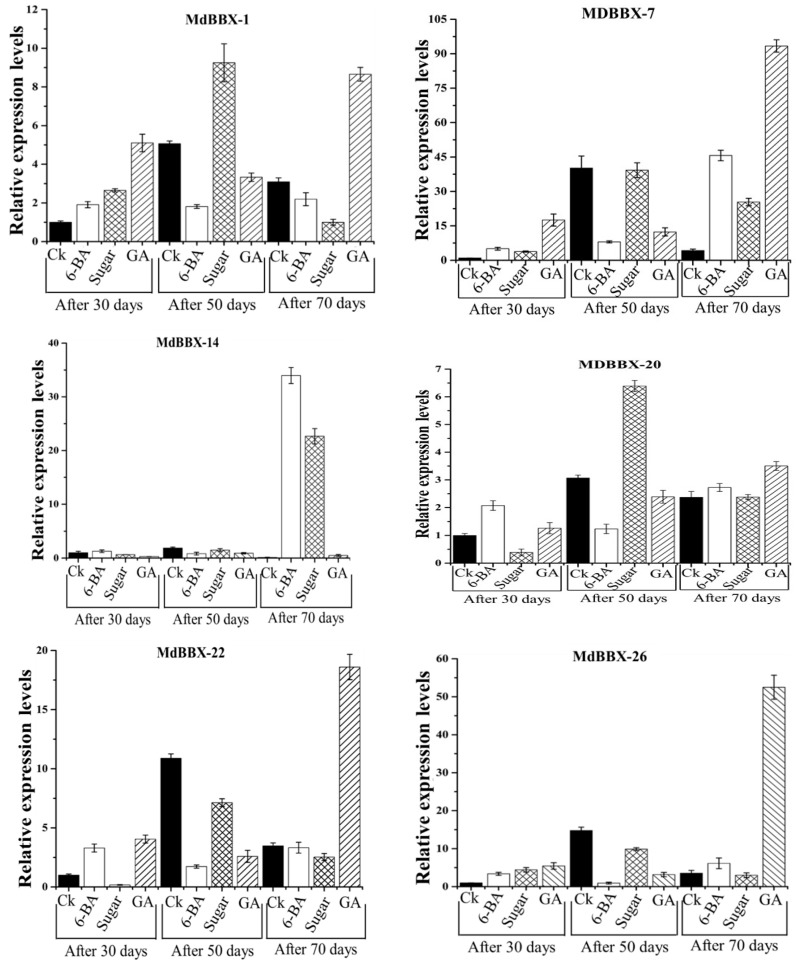

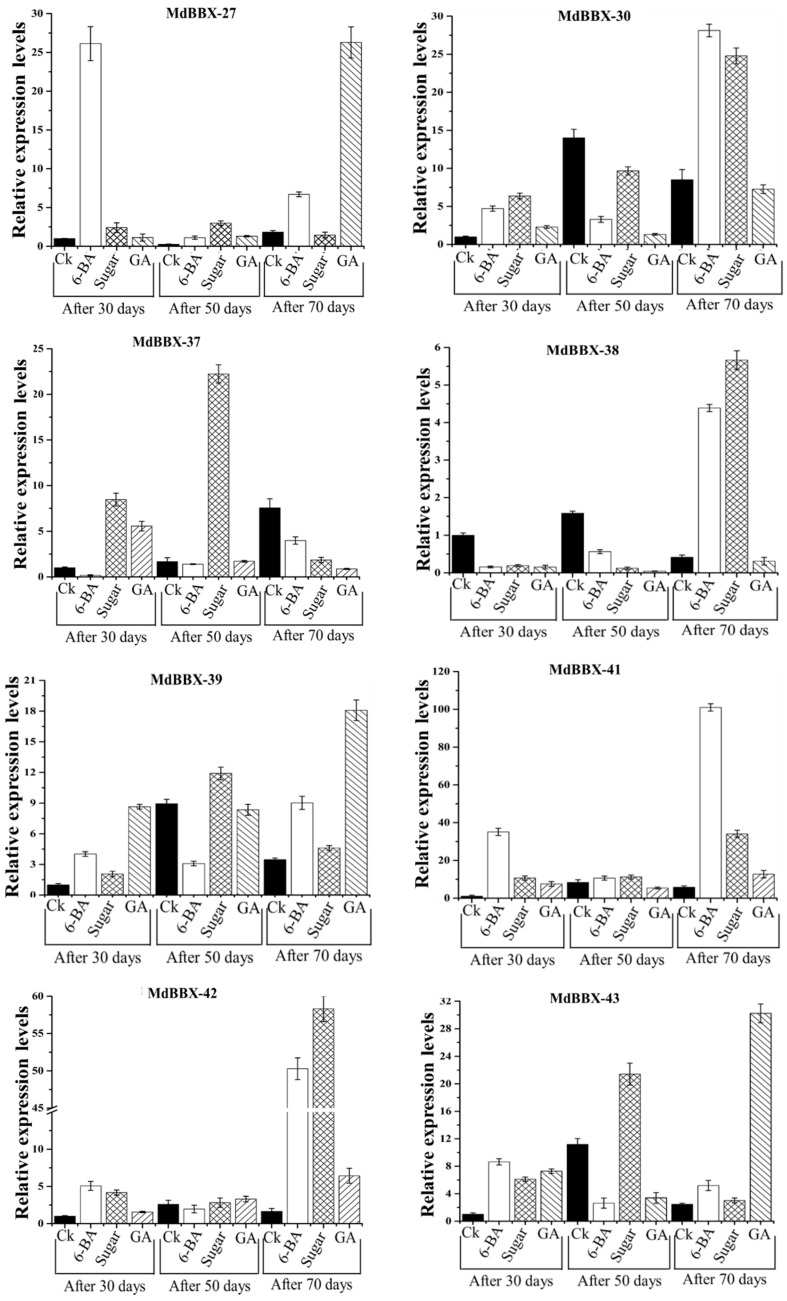

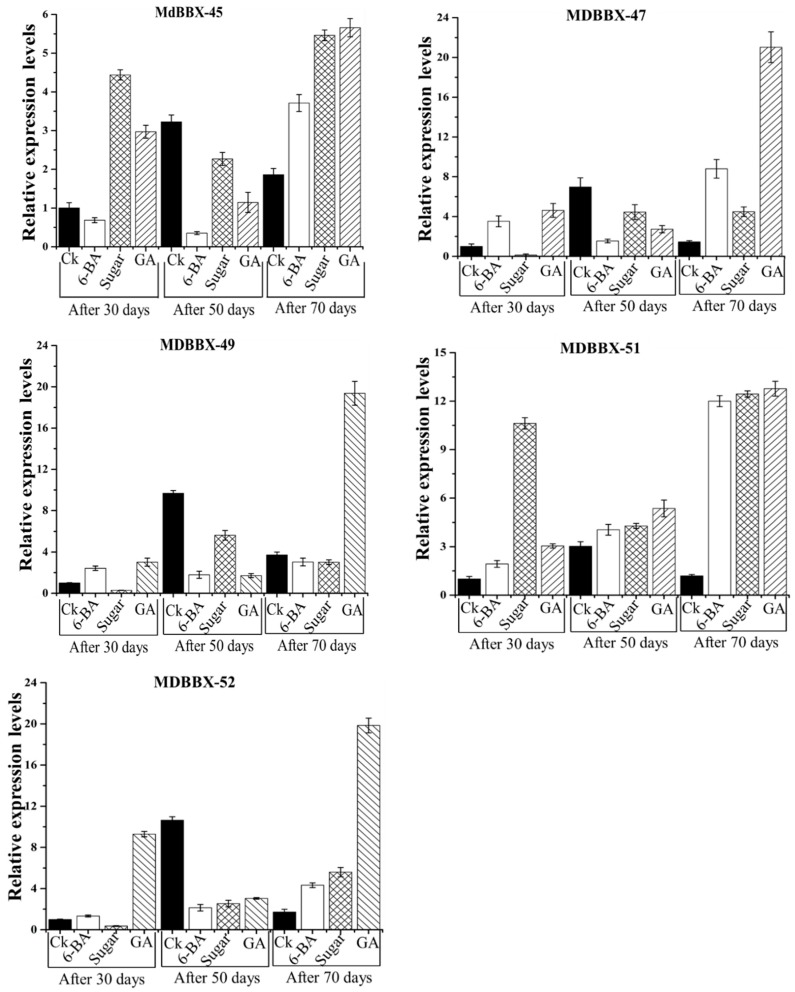
Inducible expression profile of apple *BBX* gene family members in response to flowering-related treatments (6-BA, Sugar, and GA), where Ck represents the control, 6-BA represents 6-benzylaminopurine, and GA represents gibberellins. The *x*-axis indicates the treatment. The *y*-axis shows the relative expression level of each treatment compared to control (Ck). The error bars indicate the standard deviations of the three independent qRT-PCR replicates.

Previous studies have investigated that exogenous treatment such as GA, 6-BA, and sugar can change the flowering rates. Among them, GA was reported for causing a reduction in the flowering rate, while, 6-BA and sugar enhanced the flowering rates [6,36,37]. Here we used these treatments to determine the expression pattern of 19 *MdBBX* genes in flowering-related tissues during the flowering stage. These 19 *MdBBX* genes were selected on the basis of previous transcription data [6]. The *MdBBX* genes showed diverse expression profile at different time points (Figure 9). The transcript levels of some studied *MdBBX* genes were high; while some displayed a low level of expression. The transcript levels of the studied BBX members were considerably low at 30 days after GA treatment, but higher than the control (Ck) treatment apart from *MdBBX14*, *MdBBX22*, and *MdBBX38* genes. At 50 days of GA treatment, the transcription activities of all the *BBX* gene members were downregulated as compared with the control except *MdBBX27*, *MdBBX41*, *MdBBX42*, and *MdBBX51* genes. Based on day to day analysis, we found mixed gene expression for the *BBX* gene family members. The expression patterns of almost all the *BBX* genes were similar i.e., upregulated at 30 days, then downregulated at 50 days, and then promoted at 70 days, however, we also found some BBX genes that showed different expression patterns from the rest of the *BBX* genes. For instance, the transcript level of MdBBX51 was enhanced with the passage of time, and parallel expression level was observed for *MdBBX39* at 30 and 50 days after GA inoculation. Altogether, the results noticed that the expression levels of *MdBBX* genes were high at 30 and 70 days of GA treated buds than normal buds.

In this study, the expressions of 19 *MdBBX* genes were investigated under the exogenous sugar condition (Figure 9). qRT-PCR was performed of 19 *MdBBX* gene (*MdBBX1*, *MdBBX7*, *MdBBX20*, *MdBBX22*, *MdBBX26*, *MdBBX27*, *MdBBX30*, *MdBBX37*, *MdBBX38*, *MdBBX39*, *MdBBX41*, *MdBBX42*, *MdBBX43*, *MdBBX45*, *MdBBX47*, *MdBBX49*, *MdBBX51*, and *MdBBX52*) to study the effect of exogenous sugar on the expression of *MdBBX* gene in apple. We noted that the expression of *MdBBX2*, *MdBBX7*, *MdBBX26*, *MdBBX27*, *MdBBX30*, *MdBBX37*, *MdBBX39*, *MdBBX41*, *MdBBX42*, *MdBBX43*, *MdBBX45*, and *MdBBX51* were upregulated at 30 days of sugar treatment, whereas the rest of the BBX members were downregulated compared with control (Ck) treatment. The transcribed levels for *MdBBX22*, *MdBBX26*, *MdBBX30*, *MdBBX45*, *MdBBX47*, *MdBBX49*, and *MdBBX52* were upregulated after 50 days of post treatment, but their transcript levels were lower than that of in untreated tissues. At this time point, the transcript level of *MdBBX7*, *MdBBX14*, and *MdBBX42* members were equivalent with the control treatment. Upregulation was observed for the *MdBBX7*, *MdBBX14*, *MdBBX30*, *MdBBX38*, *MdBBX39*, *MdBBX41*, *MdBBX42*, *MdBBX43*, *MdBBX45*, *MdBBX47*, *MdBBX51*, and *MdBBX52* genes at 70 days of sugar treatment. Overall, high numbers of BBX genes showed a high transcript level at all the three time points.

To gain a deeper insight into the role of *MdBBX* genes in hormonal stresses, we investigated the transcript profiles of 19 apple BBX members under 6-BA treatment (Figure 9). The plants were subjected to 6-BA treatment, where all the studied BBX members showed higher transcript levels at 30 days of treatment excluding *MdBBX37*, *MdBBX45*, and *MdBBX47* genes. The expression of 16 out of 19 *BBX* genes was lower in treated samples than untreated samples at 50 days under 6-BA treatment. The transcript level of *OsBBX27*, *MdBBX41,* and *MdBBX51* were higher than the control samples. Moreover, we noticed that the transcribed levels of *MdBBX1*, *MdBBX37,* and *MdBBX49* genes were lower than the control at 70 days treatment, whereas the expression level of remaining BBX members was promoted at this time point. Altogether, maximum genes were upregulated at 30 days, downregulated at 50 days, and then promoted at 70 days. Quite a similar transcript pattern was observed in both hormones (GA and 6-BA); however, the behaviors of *BBX* genes were different in sugar compared with GA and 6-BA. The unique inducible expression pattern of the *BBX* gene family member under hormonal and sugar treatment may indicate the role of *BBX* gene family in response to multivariate hormones and stresses. However, further studies are required to deeply investigate the particular role of *BBX* gene family in the plant under different hormones and stresses.

## 3. Materials and Methods

### 3.1. Identification of BBX Gene Family Member

The *Arabidopsis BBX* gene family has already been investigated. In the present study, all the BBX protein sequences were extracted from the *Arabidopsis* Information Resource (TAIR) database (http://www. arabidopsis.org) and were used as queries for BLASTP search with default parameters against the corresponding Genome databases. The extracted sequences proteins were manually tested for the BBX domains. All *BBX* genes were extracted automatically and manually.

### 3.2. Chromosomal Localization and Gene Duplication

Candidate *BBX* gene annotations and their chromosomal locations were obtained from the Genomic Database for *Rosaceae* (http://www.Rosaceaee.org/). The exact location of genes on chromosomes was identified by using MapDraw. The conserved and shared domains for all BBX protein sequences were created by online version 4.9.1 of the Multiple Expectation for Motif Elicitation (MEME) tool (http://meme-suite.org/) [38,39]. BBX exon-intron structure consisting of exon positions and gene length [39] were constructed through online Gene Structure Display Server (http://gsds.cbi.pku. edu.cn).

### 3.3. Sequence Alignment and Polygenetic Analysis

DNAMAN software (Version 5.2.2, LynnonBiosoft) was used to design the multiple alignments of BBX sequences. Sequence logos were generated through online Weblogo platform (http://weblogo.berkeley.edu/logo.cgi). A neighbor-joining method in MEGA 5.10 with complete deletion option was used to construct the *p*-distance-based phylogenetic tree [40].

### 3.4. Tandem Duplication and Synteny Analysis

The syntenic blocks were designed from Plant Genome Duplication Database (http://chibba.agtec.uga.edu/duplication/). Circos version 0.63 (http://circos.ca/) was used to construct the diagrams. The physical location of a gene on the chromosome was used to find out the Tandem duplication of *MdBBX* genes in apple. Genes having an adjacent homologous *BBX* gene on the same apple chromosome with no more than one intervening gene were considered to be tandemly duplicated.

### 3.5. Plant Materials and Treatments

Seventy-two uniform 6-year-old ‘Fuji’/T337/Malus robusta Rehd, apple trees were used in this present study. The studied materials were grown at the experimental orchard of Northwest Agriculture and Forestry University in Yangling (108°04′ E, 34°16′ N), China. These materials were distributed into four groups: 18 cultivars were sprayed with GA, 18 were treated with 6-BA, 18 were treated with sugar, while 18 were sprayed with water and used as a control. Every group was divided into further three blocks with three replications. The hormonal treatments were carried out with little modification in the study of Zheng et al., 2016 [29]. Briefly, 700 mg L^−^^1^ GA3 (Sigma, Deisenhofen, Germany) was sprayed once on a clear morning at 30 DAFB (days after full blooming) (9 May). Additionally, trees were sprayed with 300 mg L^−^^1^ 6-BA (Sigma) on a clear morning at 30 DAFB (9 May). The sugar treatment involved spraying trees two times with 15,000 mg L^−^^1^ and 20,000 mg L^−^^1^ sucrose on clear mornings at 30 and 37 DAFB (9 May and 16 May). All treatments involved the whole tree and the treatments were sprayed through hand wand sprayer with low pressure.

The terminal buds on current-year spurs (<5 cm) were collected into liquid nitrogen at 30, 50, and 70 DAFB. Then collected samples were stored at −80 °C to study gene expression.

### 3.6. Samples Collection

Various organs were collected for the investigation of gene expression in 6-year old Fuji’/T337/*Malus robusta* Rehd at the end of April 2017. Bud samples were collected along with mature leaves. For RNA extraction, three different samples were taken from three independent experiments for the same tissue and treatment. All the samples were immediately put into liquid nitrogen and stored at −80 °C for further study.

### 3.7. Quantitative RT-PCR Analysis

The total RNA from all the samples was extracted using the cetyltrimethylammonium bromide (CTAB) method [41]. The samples were run on 2% agarose gels to confirm the total RNA. The RNA samples were treated with RNase-free DNase to remove the residual genomic DNA. The cDNA was synthesized from all the samples. 1 μg of total RNA was used for cDNA synthesis through PrimeScript RT Reagent Kit with gDNA Eraser (Takara Bio, Shiga, Japan) following the manufacturer’s instructions. All the primers were designed from apple BBX sequences for real-time PCR using primer 6.0 (Table S3). Each primer pair was tested via standard RT-PCR to check the size specificity of the amplified product by 1% agarose gel electrophoresis. Real-time PCR was carried out in an iCycleriQ Real-Time PCR Detection System (Bio-Rad, Hercules, CA, USA). Each reaction consisted of 5 μL SYBR Premix ExTaq (Takara, Kyoto, Japan), 2 μL cDNA samples, 0.5 μL of each primer (10 μM), and 2 μLdd H_2_O in a reaction system of 10 μL. The thermal cycle was as follows: 95 °C for 3 min, followed by 40 cycles at 94 °C for 15 s, 62 °C for 20 s, and 72 °C for 20 s. Melting-curve analysis was performed directly after real-time PCR to verify the presence of gene-specific PCR products. This analysis was done at 94 °C for 15 s, followed by a constant increase from 60 to 95 °C at a 2% ramp rate. The apple *EF-1α* gene (Gen Bank accession No. DQ341381) was used as an internal control and served as a standard gene for normalizing all mRNA expression levels. The relative amount of template present in each PCR amplification mixture was evaluated by using the 2^−ΔΔ*C*t^ method, where the mean values were obtained from three independent PCR amplifications.

## 4. Discussion

Transcription factors are vital for the growth and development of plants. The *BBX* gene family is a group of transcription factor proteins possessing the B-box and some members have additional CCT conserved domains [42,43]. Genome-wide identification of the *BBX* gene family has been reported in *Arabidopsis*, rice, barley, sugar beet, grape, tomato, maize, potato, and pear [5,44,45,46,47,48,49], and is responsible for different physiological activities such as the development of flowers, seedling photomorphogenesis, shade avoidance, and responses to biotic or abiotic stresses [43]. However, no systematic study has been performed in *Rosaceae* species. Although, the *BBX* gene family has been already reported in apple and pear [34,35] we found more BBX homologs in apple and pear than previous works (Figure 1). The updated correspondent genome database could be the difference maker between our work and previously reported work on the apple and pear. Therefore, the present study aimed to identify *BBX* genes, and study the evolutionary relationships in seven *Rosaceae* species and their gene structure, chromosomal location, duplication, phylogenetic analysis, and expression pattern. We found 212 putative *BBX* genes in seven *Rosaceae* species including 67 from *Malus* × *domestica*, 40 from *Pyrus communis*, 22 from *Rosa Chinesis*, 20 from *Prunus persica*, 21 from *Fragaria vesca*, 22 from *Prunus avium*, and 20 from *Rubus occidentalis* (Figure 1).

A hypothetical model has been reported for the evolution of BBX in green plants [48]. The theory stated that originally the BBXs of green plants contained the only B-box-2 domain; however, the duplication of B-box-2 domains gave birth to the B-box-1 domain. The conserved sequence analysis showed that 27 *MdBBX* members possess two B-box domains. Similarly, we found 17, 12, 10, 11, 10, and 11 *BBX* genes with two B-boxes in *Malus* × *domestica*, *Pyrus communis*, *Rosa Chinensis, Prunus persica*, *Fragaria vesca*, *Prunus avium*, and *Rubus occidentalis* genomes, respectively, whereas the number of *BBX* genes with one B-box were greater (Figure S2). In *Arabidopsis*, 21 BBX members had two B-box domains [42]. Two B-box domains were found in 17 rice BBXs genes [30]. The number of two B-box domains containing genes was 18 in tomato [47] and potato [30], correspondingly. The occurrence of B-box sequences in different species proposed that the B-box domain is highly conserved in plants. The detail sequence alignment of *Rosaceae* BBX members (Figure S1) revealed that the B-box1 domain was highly conserved compared with the B-box2 domain, suggesting that a deletion event could happen in the B-box2 domain. It was postulated that the B-BOX2 domain of the BBX proteins is deleted in the evolution process [5].

Most of the *Rosaceae BBX* genes having two B-boxes domains were clustered into subfamily II, III, and IV. The deletion of B-box 2 leads to proteins with only the B-box 1 motif which was detected in subfamily II while remaining *BBX* genes of this category was found in subfamily V and VI.

Previous studies identified four different types of *BBX* genes based on domain organization in tomato and *Arabidopsis* [1]. We also found four different types of *BBX* genes, *BBX* genes with only one B-BOX domain, *BBX* genes with two B-boxes domains, *BBX* genes with one B-BOX, and additional CCT domains and *BBX genes* with two B-BOXes and additional CCT domains. However, we detected a small difference in the composition of various classes of *BBX* genes in different species (Figure S2). The numbers of BBX with only one B-BOX domain, two tandem B-BOXes, BOX1 plus CCT, two tandem B-boxes plus the CCT domain were 7, 8, 4, and 13, and 6, 10, 5, and 8 in *Arabidopsis* and tomato, respectively, however this arrangement was 33, 16, 8, and 10 in apple, 13, 13, 3, and 11 in pear, 5, 5, 4, and 6 in peach, 7, 6, 3, and 5 in strawberry, 6, 7, 4, and 5 in Rose, 10, 4, 2, and 6 in sweet cherry, and 6, 6, 3, and 5 in black raspberry. The results indicate that *BBX* gene family may share conserved gene architecture and domain organization in plants during the evolution process.

The study of BBX in *Arabidopsis* [42] divided the 32 BBX candidates into five subfamilies on the basis of conserved domains. Subfamily 1 and II contained 13 *AtBBX* members (*AtBBX* to *AtBBX13*), and these genes had two B-BOXes and additional CCT domains. *AtBBX14* to *AtBBX17* carries BOX1 and the CCT domains which were clustered into subfamily III. *AtBBX18* to *AtBBX25* were grouped to subfamily IV, and possess both B-BOX1 and B-BOX2 domains. The remaining *AtBBX* genes (*AtBBX26* to *AtBBX32*) held the B-BOX1 domain, and were only noticed in subfamily V. However, conserved domains based classification of *Rosaceae* BBX proteins was rather difficult. We observed that the clustering phenomena of *Rosaceae* species BBX genes were different from the *Arabidopsis* BBX (Figure 4). A maximum number of double B-Box and additional CCT domain containing BBX members were detected in V and VI subfamilies, whereas in *Arabidopsis* these *BBX* genes were clustered together in subfamily I and II. One B-box and additional CCT domain possessing *Rosaceae* BBX members were noted in subfamily I, V, and VI, whereas such type of BBX members was found in subfamily III. A high number of only one B-box domain containing BBX members were noticed in subfamily II and V in *Rosaceae* and *Arabidopsis*, respectively. A similar difference was observed for BBX members having two B-box domains between *Rosaceae* and *Arabidopsis*. The reason behind the uneven distribution may be due to a large number of genes or small differences in the domain organization among the plant species. For instance, we noticed that 7 *BBX* genes possessed only one B-BOX domain, 8 *BBX* gene members had two B-boxes domain, 4 BBX members contained one B-BOX and an additional CCT domain, and 13 *BBX* genes were found with two B-BOXes and additional CCT domains in *Arabidopsis*. On the contrary, 33 *BBX* genes possessed only one B-BOX domain, 16 *BBX* genes found having two B-boxes domains, one B-BOX and additional CCT domain were observed in 8 BBX members and 10 *BBX* genes comprised of two B-BOXes and additional CCT domains in apple *BBX* genes. Similar differences were also observed for B-BOX genes in other *Rosaceae* members in the present study. However, we noticed that most of the members shared similar gene structures and functions within the subfamily. We also found some *BBX* genes members that showed different intron-exon structure within the same subfamily, but they were similar in function. For instance, *MdBBX14* (two introns and three exons) and *MdBBX41* (three introns and four exons) showed similar expression patterns in tested samples. We also observed some BBX members that were similar in structure showed different functions such as *MdBBX41* (three introns and four exons) and *MdBBX43* (three introns and four exons).

Gene duplication could be tandem, segmental, or be caused by something from the whole genome, and duplicated genes are important for the plant growth and development. Recently, various genes encoding transcription factors have been recognized, and most of them showed gene duplication such as AP2 (ethylene-responsive-element-binding AP2 DNA-binding domain), MADS, SPL, and DOF (DNA binding with One Finger) [30,32,49,50]. Gene duplication phenomena are vital for gene family expansion. The expansion and duplication analysis of *BBX* genes have also been carried out in maize [46] and rice [8]. Eight pairs of *MdBBX* were found to participate in tandem duplication, while thirty-seven pairs were observed as segmental duplicated in this study. These findings suggest that the expansion of the *BBX* gene family in apple occurred through tandem and segmental duplication. The quite similar mechanism of gene family evolution was also found in the GRAS (Gibberellic acid insensitive, repressor, and scarecrow) gene family in apple [51].

The identified *BBX* genes were distributed unevenly in the whole genome of studied species. The highest numbers of genes were found on chromosome 13, 1, 2, 6, 6, 3, and 2 in apple, peach, black raspberry, strawberry, sweet cherry, and Rose genomes, respectively (Figure 2). The chromosomal appearance of *BBX* genes was different from studied *BBX* genes in other species. No *BBX* gene was observed on chromosome 4 in apple, similar findings were previously found in pear [47]. In addition, fourteen *MdBBXs* were not mapped on the chromosome, maybe due to the high level of heterozygosity or quality of apple genome sequence. We used the ArrayExpress data to study the expression pattern of the *MdBBX* genes in different tissues and organs to determine the potential function of the *BBX* gene family in the growth and development of apple (Figure 7). The diverse expression pattern of apple BBX genes may be due to gene location, length, and sequences. We studied the transcript level of these genes in five different tissues by qRT-PCR based on the expression patterns of *MdBBX* genes obtained from microarray database. The *MdBBX* genes displayed different expression patterns among the tested tissues. Leaves and buds were used to study flower induction in *Arabidopsis* and other plants [52]. Most of the studied *MdBBX* genes have higher expression levels in leaf and buds compared with other tissues, showing their influence in the flower induction (Figure 8). Additionally, the transcribed level of *MdBBX* genes was considerably similar with the results from microarray data. The present study suggested that some *MdBBX* genes with diverse expression profiles in examined tissues might play vital roles in plant development, and some *MdBBX* genes may have a unique function in different developmental stages.

Several environmental and internal factors such as temperature, hormones, and photosynthesis control flower induction in plants [6,9,16]. Hormones and sugar alter the flowering in apple, however, the relationship of *MdBBX* with hormones and sugar remains unknown. Up to date, GA, 6-BA, and sugar are considered to be associated with flower induction. Previous studies also found that exogenous treatment of (GA3, 6-BA, and sugar) can change the flowering rates [6,36,37]. Although the contribution of *BBX* genes in the regulation of plant growth and development have been well studied, little is known regarding the involvement of *BBX* genes in the flower induction in fruit species. Several investigations have proposed that *BBX* genes are important for the photoperiodic regulation of flowering, seedling photomorphogenesis, shade avoidance, and responses to biotic and abiotic stresses. However, it has been also stated that CO encodes a putative zinc finger transcription factor that is key for the promotion of flowering [53]. Some BBXs (BBX32, COL6-8, 16/BBX14-17, and BBX7) controlled the flowering time in Chrysanthemum [8]. Based on the previous studies, we evaluated the expression of *MdBBX* genes in response to these flowering-related treatments. The members of the apple *BBX* gene family showed distinct expression level after treatments. Previous studies also investigated that *BBX* genes are involved in flowering and hormones response. *AtBBX1* of *Arabidopsis* promotes flowering during long days [20,51]. *AtBBX18* controls gibberellin homeostasis in *Arabidopsis* [38]. Some BBXs such as BBX2 and BBX3 have little effect on flowering time [22]. Here we found that *MdBBXs* genes showed diverse gene expression under hormones at a different time point in flowering buds (Figure 9). The changes in expression level of apple BBX genes in flowering-related buds indicates their involvement in flowering induction. A similar expression was displayed by the IDD and GRAS gene family in apple [50,54], and it was postulated that their diverse transcription profile during flower induction showed that they may involve in GA-mediated, 6-BA mediated, or sugar-mediated flowering in apple trees. We also found that some *MdBBXs* belong to the same phylogenetic group (*MdBBX37*-*MdBBX38*) which had a distinct expression profile under treatments indicating that these genes obtained a unique expression profile during evolution. These results were supported by the study of BBX in rice and tomato [40,45], which suggested that phylogenetic clustering is not highly related to functional grouping.

In conclusion, this investigation found 212 *BBX* genes in the seven members of the *Rosaceae* species which were distributed unevenly in the whole genome. We classified these *BBX* genes and performed phylogenetic, structural, synteny, and functional analysis. The present study examined the expression pattern of 19 apple BBX members in different tissues (bud, stem, leaves, flowers, and fruits). The spatiotemporal transcription level indicated that they control multiple functions of plant growth and development. Additionally, the transcript level of some *MdBBX* members was evaluated under treatments with several flowering-related compounds which suggested that some members of *MdBBXs* play a vital role in the complex flower induction process. These results may be fruitful for the improvement of flower induction in apple.

## Figures and Tables

**Table 1:** The sequence analysis and weblog of 10 identified motifs of the *BBX* gene family in seven *Rosaceae* species.

S. No.	Sequences	Width	Logo
1	HSANKLASRHQRVLLCHVSS	66	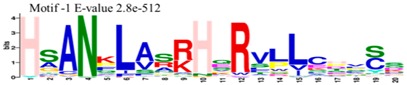
2	PARVYCKADEAALCVACDAKV	67	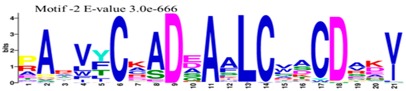
3	KKTRKFEKTIRYASRKAYAETRPRIKGRF	21	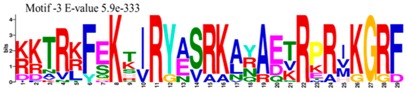
4	CDICQEAAAFLFCREDRALLCRECDAIIHAANKRTSKHERF	16	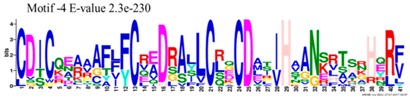
5	SPTPWKGSGNKLGPTVSVCESCVNNLEGRHEEDEDEDDDDE	14	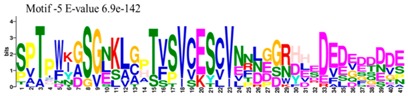
6	WLIPNPNFNSKLHMDIAPDILKSSDDLIFPEIDSLLEFDYPTSVHTISGS	5	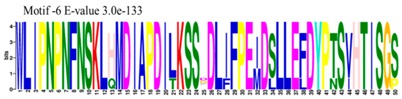
7	DDGAEEDEENQVVPLSSTPPPPPPSSSASSREZRLSESVNGDGSSARREP	8	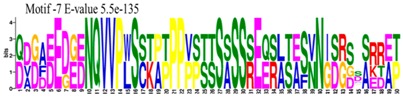
8	PLGDGPGIQMPTQLSDMDREARVLRYREK	15	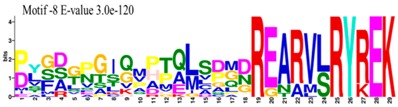
9	PVQPDPIPPPSFNMNYNISGPADHNCFDLDFCRSKLYSSFN	5	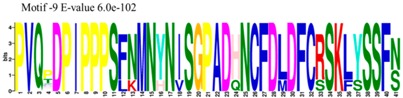
10	MKIQCDSCZKA	45	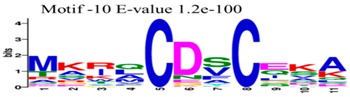

## Supplementary Materials

Supplementary Materials are available online.

## Abbreviations

Ck	control
GA	gibberellins;
6-BA	6-benzylaminopurine;
DAFB	days after full bloom
