# Supplementary material for: Genome Identification of B-BOX Gene Family Members in Seven Rosaceae Species and Their Expression Analysis in Response to Flower Induction in Malus domestica
Source: Molecules. 2018 Jul 18;23(7):1763. doi: 10.3390/molecules23071763 (PMC6100437; doi:10.3390/molecules23071763)
Supplement: Supplementary file 1 [file molecules-23-01763-s001.pdf]

Table S1: Nomenclauture, Identification, chromosomal location, CDS and peptide length and weight of BBX gene family in seven Rosacea species.

Name	Gene Id	Location	CDS	Molecular weight(KD)	Peptide
MdBBX1	MDP0000202669	chr1:26862700..26863811	1023	38.14	340
MdBBX2	MDP0000294359	chr2:19905176..19906517	1224	44.10	407
MdBBX3	MDP0000172036	chr3:28997567..28999608	450	16.32	149
MdBBX4	MDP0000820175	chr3:31899712..31900798	375	13.83	124
MdBBX5	MDP0000315396	chr3:33580850..33589540	780	29.71	259
MdBBX6	MDP0000316084	chr3:33595541..33598751	804	30.46	267
MdBBX7	MDP0000289278	chr5:2765013..2765447	435	15.89	144
MdBBX8	MDP0000939920	chr5:2779394..2779828	435	15.89	144
MdBBX9	MDP0000761905	chr5:2792867..2793301	435	15.89	144
MdBBX10	MDP0000598183	chr5:5786734..5786946	213	7.69	70
MdBBX11	MDP0000280947	chr5:20737133..20745836	2577	94.70	858
MdBBX12	MDP0000140460	chr5:20744637..20745869	546	20.25	181
MdBBX13	MDP0000733075	chr6:16920878..16921438	561	20.68	186
MdBBX14	MDP0000271388	chr6:22904175..22906599	1341	49.54	446
MdBBX15	MDP0000283949	chr7:11349283..11353918	1458	53.05	485
MdBBX16	MDP0000242475	chr7:14787088..14787441	354	12.80	117
MdBBX17	MDP0000264228	chr7:25836126..25837798	1374	49.79	457
MdBBX18	MDP0000264845	chr7:25837817..25839485	1443	52.27	480
MdBBX19	MDP0000551876	chr8:1381940..1386799	1335	49.06	444
MdBBX20	MDP0000297093	chr9:4850373..4852477	1179	43.56	392
MdBBX21	MDP0000194889	chr9:6486783..6487555	693	24.71	230
MdBBX22	MDP0000198531	chr9:6495007..6495595	558	20.07	185
MdBBX23	MDP0000232355	chr9:6833588..6836630	1371	49.64	456
MdBBX24	MDP0000177126	chr9:9619025..9620952	1248	40.52	415
MdBBX25	MDP0000587860	chr9:12133412..12133600	189	6.74	62
MdBBX26	MDP0000222881	chr9:24121313..24122788	990	35.22	329
MdBBX27	MDP0000800387	chr10:10605557..10606492	627	23.26	208
MdBBX28	MDP0000901915	chr11:33378342..33379421	1080	39.41	359
MdBBX29	MDP0000208320	chr11:33997775..34000066	996	36.57	331
MdBBX30	MDP0000915501	chr12:21541142..21542428	909	33.25	302
MdBBX31	MDP0000383112	chr13:2085719..2086194	192	7.10	63
MdBBX32	MDP0000131980	chr13:2544446..2546146	1365	50.48	454
MdBBX33	MDP0000314259	chr13:2562817..2564517	1365	50.48	454
MdBBX34	MDP0000247810	chr13:2563965..2564201	237	8.57	78
MdBBX35	MDP0000458656	chr13:2569771..2570873	558	20.55	185
MdBBX36	MDP0000697407	chr13:3060206..3061697	906	32.35	301
MdBBX37	MDP0000302297	chr13:8847122..8862470	2772	101.43	923
MdBBX38	MDP0000565292	chr13:15864540..15876017	1980	71.94	659
MdBBX39	MDP0000151848	chr13:33621832..33624451	759	27.98	252

MdBBX40	MDP0000157816	chr14:9991925..9992740	816	29.64	271
MdBBX41	MDP0000273201	chr14:27661064..27667646	2220	83.53	741
MdBBX42	MDP0000298575	chr16:774671..782658	2307	86.28	768
MdBBX43	MDP0000321380	chr16:1224658..1229786	1752	64.67	583
MdBBX44	MDP0000185616	chr16:1224811..1226412	1362	50.29	453
MdBBX45	MDP0000713113	chr16:20659246..20667253	1404	50.37	467
MdBBX46	MDP0000140484	chr17:5396728..5398846	1182	43.56	393
MdBBX47	MDP0000298635	chr17:5396890..5398976	1182	43.56	393
MdBBX48	MDP0000241871	chr17:5399009..5401138	828	30.91	275
MdBBX49	MDP0000321180	chr17:6874441..6877832	945	34.65	314
MDBBX50	MDP0000198072	chr17:6874660..6875470	729	26.37	242
MdBBX51	MDP0000782323	chr17:9847401..9849311	1230	44.49	409
MdBBX52	MDP0000759984	chr17:12792679..12793491	813	29.42	270
MdBBX53	MDP0000398010	chr17:20057435..20058249	603	21.97	200
MdBBX54	MDP0000128008	unanchored:7356965..7358489	609	22.77	202
MdBBX55	MDP0000664576	unanchored:12855188..12856983	732	26.66	243
MdBBX56	MDP0000321735	unanchored:12861348..12862789	561	20.48	187
MdBBX57	MDP0000189746	unanchored:24652531..24654252	1371	50.30	456
MdBBX58	MDP0000127949	unanchored:30937667..30939034	1062	38.78	353
MdBBX59	MDP0000232445	unanchored:34963592..34966330	900	32.93	299
MdBBX60	MDP0000313949	unanchored:63077159..63081560	1920	72.36	639
MdBBX61	MDP0000686172	unanchored:64578669..64579103	435	15.89	144
MdBBX62	MDP0000128581	unanchored:66464234..66465313	1080	39.41	359
MdBBX63	MDP0000244238	unanchored:66464516..66465595	1080	39.41	359
MdBBX64	MDP0000272743	unanchored:74236542..74238208	1368	49.59	455
MdBBX65	MDP0000488955	unanchored:76682982..76683170	189	6.70	62
MdBBX66	MDP0000697109	unanchored:100056446..100056917	237	8.35	78
MdBBX67	MDP0000122414	unanchored:116968393..116968930	366	12.55	121
		(Pear, Pyrus *communis*)			
PcBBX1	PCP009714.1	scaffold00007:169646..171470	1356	49.37	452
PcBBX2	PCP043963.1	scaffold00007:519721..521820	1398	51.07	466
PcBBX3	PCP016214.1	scaffold00011:692760..694164	1020	38.73	340
PcBBX4	PCP035867.1	scaffold00014:563819..564244	423	15.92	141
PcBBX5	PCP005095.1	scaffold00024:549462..550506	810	29.32	270
PcBBX6	PCP022591.1	scaffold00035:232464..233907	906	33.34	302
PcBBX7	PCP014647.1	scaffold00070:241159..249737	504	18.25	168
PcBBX8	PCP024261.1	scaffold00076:130476..133225	1161	42.36	387
PcBBX9	PCP036200.1	scaffold00095:68171..76508	2784	101.6	928
PcBBX10	PCP011581.1	scaffold00128:258610..260887	1194	44.38	398
PcBBX11	PCP030625.1	scaffold00180:63246..66485	393	13.10	131
PcBBX12	PCP030637.1	scaffold00180:214739..216317	870	31.94	290
PcBBX13	PCP005365.1	scaffold00204:137886..139947	603	22.89	201
PcBBX14	PCP005445.1	scaffold00264:269670..275747	1932	72.89	644

PcBBX15	PCP016580.1	scaffold00271:22086..23080	744	26.03	248
PcBBX16	PCP042842.1	scaffold00317:112198..114125	1245	45.99	415
PcBBX17	PCP005533.1	scaffold00364:92726..95120	1074	39.06	358
PcBBX18	PCP033159.1	scaffold00365:188908..191817	1374	50.82	458
PcBBX19	PCP016741.1	scaffold00411:124538..125217	441	16.47	147
PcBBX20	PCP018295.1	scaffold00452:206496..207931	903	32.93	301
PcBBX21	PCP039894.1	scaffold00467:78987..81027	666	24.88	222
PcBBX22	PCP021617.1	scaffold00594:143726..146870	780	27.59	260
PcBBX23	PCP023237.1	scaffold00635:23866..29654	1245	45.47	415
PcBBX24	PCP044704.1	scaffold00702:47152..50034	891	32.21	297
PcBBX25	PCP028057.1	scaffold00738:58516..63937	1689	61.37	563
PcBBX26	PCP012120.1	scaffold00748:102457..103657	813	29.73	271
PcBBX27	PCP007365.1	scaffold00765:1816..2690	738	26.42	246
PcBBX28	PCP015430.1	scaffold00990:92630..93637	564	21.85	188
PcBBX29	PCP009084.1	scaffold01146:64216..65974	1230	44.98	410
PcBBX30	PCP010729.1	scaffold01267:21807..27157	720	25.79	240
PcBBX31	PCP017205.1	scaffold01311:34630..35897	1020	37.49	340
PcBBX32	PCP028298.1	scaffold01398:71203..78157	615	22.81	205
PcBBX33	PCP014014.1	scaffold01509:68826..74781	1293	48.10	431
PcBBX34	PCP022040.1	scaffold01674:47276..48600	1008	36.11	336
PcBBX35	PCP035132.1	scaffold01811:37244..38937	1323	49.13	441
PcBBX36	PCP026940.1	scaffold02017:12727..25097	3273	121.1	1091
PcBBX37	PCP025314.1	scaffold02096:136..1889	1362	50.17	454
PcBBX38	PCP045197.1	scaffold05997:857..2571	597	21.90	199
PcBBX39	PCP022283.1	scaffold07834:1367..3297	726	26.88	242
PcBBX40	PCP040624.1	scaffold17349:177..2057	921	33.13	307
		Rosa Chinesis (Rose)			
RcBBX1	RC0G0003800	Chr00:350774..354991	1251	45.19	417
RcBBX2	RC0G0110200	Chr00:13204519..13205726	1074	39.09	358
RcBBX3	RC0G0138600	Chr00:17414184..17416216	717	26.53	239
RcBBX4	RC2G0139900	Chr02:11653012..11655635	561	20.34	187
RcBBX5	RC2G0315700	Chr02:33096974..33097822	639	22.80	213
RcBBX6	RC2G0491300	Chr02:57402185..57405076	696	25.77	232
RcBBX7	RC2G0501900	Chr02:58418265..58419568	792	28.46	264
RcBBX8	RC2G0550600	Chr02:63202976..63205538	1350	49.28	450
RcBBX9	RC2G0550700	Chr02:63205797..63207626	270	10.74	90
RcBBX10	RC2G0558900	Chr02:64087302..64088912	765	28.49	255
RcBBX11	RC2G0585400	Chr02:66344092..66346528	882	32.77	294
RcBBX12	RC4G0386100	Chr04:49891627..49894003	1359	51.58	453
RcBBX13	RC4G0385900	Chr04:49862326..49862904	576	20.01	192
RcBBX14	RC4G0386000	Chr04:49863689..49867711	1041	38.42	347

RcBBX15	RC4G0394000	Chr04:50602599..50604335	1338	49.92	446
RcBBX16	RC5G0261500	Chr05:22136743..22140055	855	30.09	285
RcBBX17	RC5G0319200	Chr05:29061736..29062603	723	26.01	241
RcBBX18	RC6G0195500	Chr06:22416546..22417889	648	24.34	216
RcBBX19	RC6G0423000	Chr06:53394265..53397720	1167	43.11	389
RcBBX20	RC6G0448100	Chr06:55381121..55382633	1203	44.00	401
RcBBX21	RC6G0508800	Chr06:60256429..60257878	891	32.19	297
RcBBX22	RC7G0081000	Chr07:5581172..5583667	1539	57.77	513
		Prunus Avioum (Sweet Cherry)			
PaBBX1	Pav_sc0004527.1_g080.1.mk	PAV_r1.0chr1:17737787..17743220	717	26.43	239
PaBBX2	Pav_sc0001051.1_g030.1.mk	PAV_r1.0chr1:24564167..24566102	999	35.19	333
PaBBX3	Pav_sc0001181.1_g240.1.mk	PAV_r1.0chr1:24997238..24998521	1950	71.18	650
PaBBX4	Pav_sc0000131.1_g850.1.mk	PAV_r1.0chr1:29989611..29991028	1350	49.26	450
PaBBX5	Pav_sc0002456.1_g010.1.mk	PAV_r1.0chr1:43036607..43051043	891	33.28	297
PaBBX6	Pav_sc0000293.1_g410.1.mk	PAV_r1.0chr3:661262..662056	1029	38.77	343
PaBBX7	Pav_sc0000592.1_g560.1.mk	PAV_r1.0chr3:11351478..11353057	1644	59.04	548
PaBBX8	Pav_sc0000488.1_g110.1.mk	PAV_r1.0chr3:14498374..14502886	792	28.08	264
PaBBX9	Pav_sc0000405.1_g160.1.mk	PAV_r1.0chr3:16146055..16148421	672	24.55	224
PaBBX10	Pav_sc0000405.1_g200.1.mk	PAV_r1.0chr3:16170043..16170396	1254	45.29	418
PaBBX11	Pav_sc0001859.1_g200.1.mk	PAV_r1.0chr3:16934620..16935833	1197	42.79	399
PaBBX12	Pav_sc0000051.1_g110.1.mk	PAV_r1.0chr3:18112949..18115389	351	13.82	117
PaBBX13	Pav_sc0000598.1_g150.1.mk	PAV_r1.0chr4:1931059..1932064	456	17.06	152
PaBBX14	Pav_sc0000352.1_g070.1.mk	PAV_r1.0chr4:11752866..11755126	972	35.87	324
PaBBX15	Pav_sc0000124.1_g140.1.mk	PAV_r1.0chr4:14560239..14561009	612	23.58	204
PaBBX16	Pav_sc0000383.1_g410.1.mk	PAV_r1.0chr5:15713586..15715861	900	31.31	300
PaBBX17	Pav_sc0000044.1_g810.1.mk	PAV_r1.0chr7:13871819..13874309	576	21.37	192
PaBBX18	Pav_sc0000175.1_g260.1.mk	PAV_r1.0chr8:8049172..8053512	1440	53.68	480
PaBBX19	Pav_sc0001518.1_g660.1.mk	PAV_r1.0chr8:8383526..8384850	687	25.08	229
PaBBX20	Pav_sc0000848.1_g470.1.mk	PAV_r1.0chr8:19897720..19900725	546	20.75	182
PaBBX21	Pav_sc0004527.1_g080.1.mk	PAV_r1.0chr1:17737787..17743220	624	23.66	208
PaBBX22	Pav_sc0001051.1_g030.1.mk	PAV_r1.0chr1:24564167..24566102	1173	43.97	391
		Rubus *occidentalis (*Black raspberry*)*			
RoBBX1	Bras_T01148	chr1_v1.1:19757119..19760254	747	27.66	249
RoBBX2	Bras_T23037	chr2_v1.1:5029153..5033176	624	23.86	208
RoBBX3	Bras_T14233	chr2_v1.1:5300809..5302758	645	24.04	215
RoBBX4	Bras_T02515	chr2_v1.1:9603227..9604828	1017	37.33	339
RoBBX5	Bras_T02294	chr2_v1.1:10747166..10748922	900	32.76	300
RoBBX6	Bras_T07074	chr2_v1.1:17881853..17884695	1185	43.45	395
RoBBX7	Bras_T18105	chr3_v1.1:20008409..20009743	741	27.93	247
RoBBX8	Bras_T18591	chr3_v1.1:23954027..23956321	846	30.88	282
RoBBX9	Bras_T05889	chr3_v1.1:31762193..31767034	684	25.37	228

RoBBX10	Bras_T02680	chr4_v1.1:4650595..4652026	1302	48.18	434
RoBBX11	Bras_T23114	chr4_v1.1:7464873..7467556	1437	52.30	479
RoBBX12	Bras_T25113	chr4_v1.1:11937754..11939524	1095	40.34	365
RoBBX13	Bras_T16297	chr5_v1.1:3211002..3213352	1359	50.09	453
RoBBX14	Bras_T03753	chr6_v1.1:228708..229566	648	23.07	216
RoBBX15	Bras_T04853	chr6_v1.1:4550295..4552732	1188	43.48	396
RoBBX16	Bras_T23364	chr6_v1.1:5339364..5340269	795	28.96	265
RoBBX17	Bras_T22204	chr6_v1.1:5605179..5605529	348	13.29	116
RoBBX18	Bras_T22203	chr6_v1.1:5607139..5609397	1359	49.44	453
RoBBX19	Bras_T26276	chr6_v1.1:15029911..15032044	546	19.54	182
RoBBX20	Bras_T24206	chr6_v1.1:15618605..15619375	768	27.22	256
		Prunus Persica (Peach)			
PpBBX1	ppa007083m	scaffold_1:6788512..6789796	1149	41.82	383
PpBBX2	ppa010704m	scaffold_1:17830935..17834763	717	26.54	239
PpBBX3	ppa004756m	scaffold_1:22447084..22450267	1479	53.48	493
PpBBX4	ppa005713m	scaffold_1:29244300..29245928	1341	49.78	447
PpBBX5	ppa022195m	scaffold_1:29624131..29626487	1380	51.78	460
PpBBX6	ppa011525m	scaffold_1:32859280..32860179	621	22.30	207
PpBBX7	ppa008143m	scaffold_1:34301935..34303374	1029	38.51	343
PpBBX8	ppa026514m	scaffold_3:611907..612413	507	17.98	169
PpBBX9	ppa010493m	scaffold_3:12066729..12068386	741	27.48	247
PpBBX10	ppa006303m	scaffold_3:15262568..15266469	1254	45.27	418
PpBBX11	ppa025622m	scaffold_3:16815877..16818464	1353	49.75	451
PpBBX12	ppa022228m	scaffold_3:16836056..16836409	351	13.94	117
PpBBX13	ppa009942m	scaffold_3:17290319..17291325	813	29.29	271
PpBBX14	ppa007007m	scaffold_3:18343501..18345686	1158	42.31	386
PpBBX15	ppa010671m	scaffold_4:11588344..11589492	720	26.90	240
PpBBX16	ppa017267m	scaffold_5:17214327..17217139	1320	49.99	440
PpBBX17	ppa012724m	scaffold_7:17379028..17381072	471	17.07	157
PpBBX18	ppa011488m	scaffold_8:11063691..11065316	624	22.95	208
PpBBX19	ppa026391m	scaffold_8:11362565..11363292	624	23.75	208
PpBBX20	ppa006893m	scaffold_8:20983651..20985809	1173	43.28	391
		Fragaria vesca (Strawberry)			
FvBBX1	FvH4_1g12110.1	Fvb1_v4.0.a1:6615402..6618534	558	20.20	186
FvBBX2	FvH4_2g10070.1	Fvb2_v4.0.a1:8897464..8898927	636	23.62	212
FvBBX3	FvH4_2g24910.1	Fvb2_v4.0.a1:20252285..20254597	1221	45.37	407
FvBBX4	FvH4_2g38990.1	Fvb2_v4.0.a1:28109209..28111377	939	34.57	313
FvBBX5	FvH4_2g41420.1	Fvb2_v4.0.a1:29305066..29306602	936	34.73	312
FvBBX6	FvH4_3g03640.1	Fvb3_v4.0.a1:2045334..2045885	549	20.61	183
FvBBX7	FvH4_3g17750.1	Fvb3_v4.0.a1:11299511..11301015	861	30.6	287
FvBBX8	FvH4_3g21230.1	Fvb3_v4.0.a1:14224437..14225826	741	27.5	247

FvBBX9	FvH4_4g08980.1	Fvb4_v4.0.a1:10078078..10079648	1068	38.97	356
FvBBX10	FvH4_4g10930.1	Fvb4_v4.0.a1:14597113..14598985	714	26.62	238
FvBBX11	FvH4_4g23090.1	Fvb4_v4.0.a1:25667550..25687393	3483	126.05	1161
FvBBX12	FvH4_4g26540.1	Fvb4_v4.0.a1:27897258..27898373	489	17.56	163
FvBBX13	FvH4_4g26550.1	Fvb4_v4.0.a1:27899035..27902637	1413	52.81	471
FvBBX14	FvH4_4g27390.1	Fvb4_v4.0.a1:28409275..28411534	1350	50.90	450
FvBBX15	FvH4_5g12150.1	Fvb5_v4.0.a1:6852197..6854626	1371	51.43	457
FvBBX16	FvH4_6g37140.1	Fvb6_v4.0.a1:29197326..29198120	792	28.63	264
FvBBX17	FvH4_6g37790.1	Fvb6_v4.0.a1:29751710..29755900	210	7.93	70
FvBBX18	FvH4_6g40380.1	Fvb6_v4.0.a1:31931440..31935769	1251	45.46	417
FvBBX19	FvH4_6g43570.1	Fvb6_v4.0.a1:33682892..33686067	1332	48.65	444
FvBBX20	FvH4_6g43580.1	Fvb6_v4.0.a1:33686668..33687006	336	12.81	112
FvBBX21	FvH4_6g44270.1	Fvb6_v4.0.a1:34125442..34128499	756	27.25	252

Table S2: The detail information about physiochemical characteristics of BBX Gene family in seven *Rosacea* species.

Name	Isoelectric Point	Instability Index	GRAVY	Major Amino Acid[a]	Aliphatic Index	Strand
MdBBX1	5.74	48.93	-0.571	A(10.6%)、S(10%)、V(7.4%)	65.18	+
MdBBX2	8.11	47.83	-0.295	S(10.8%)、A(10.6%)、P(7.9%)	71.47	-
MdBBX3	5.35	45.68	-0.372	L(10.7%)、G(9.4%)、A(8.1%)	74.63	-
MdBBX4	6.55	40.02	-0.128	L(10.5%)、G(8.9%)、R(8.1%)	76.21	+
MdBBX5	6.75	51.71	0.62	L(9.3%)、V(8.9%)、P(8.5%)	95.56	-
MdBBX6	9.53	49.57	-0.203	V(9.0%)、L(8.6%)、P(8.2%)	84.64	+
MdBBX7	4.58	69.35	-0.791	S(13.2%)、D(9.7%)、E(9.7%)	56.11	+
MdBBX8	4.58	69.35	-0.791	S(13.2%)、D(9.7%)、E(9.7%)	56.11	+
MdBBX9	4.58	69.35	-0.791	S(13.2%)、D(9.7%)、E(9.7%)	56.11	+
MdBBX10	7.50	27.50	0.187	A(12.9%)、C(12.9)、L/ K(10%)	94.86	+
MdBBX11	6.13	41.54	-0.173	L(8.9%)、G(8.3%)、S(7.7%)	82.49	-
MdBBX12	8.41	60.04	-0.618	A(9.4%)、R(8.3%)、P(7.7%)	66.85	+
MdBBX13	4.55	49.07	-0.864	E(12.9%)、D(9.7%)、S(9.7%)	52.96	+
MdBBX14	5.21	54.60	-0.574	A /E(9.8%)、G /L(7.8%)	71.79	-
MdBBX15	7.14	44.83	-0.449	A(9.7%)、G(8.5%)、L(8.2%)	65.67	-
MdBBX16	8.88	57.75	-0.342	A(12%)、S(11%)、P /T(8.5%)	60.94	+
MdBBX17	6.54	48.50	-0.215	S(10.7%)、A(9.8%)、L(8.5%)	75.21	+
MdBBX18	8.44	47.81	-0.219	S(10.6%)、A(10.4%)、L(8.8%)	78.31	+
MdBBX19	6.28	49.36	-0.381	L(9.0%)、S(8.8%)、A(7.9%)	71.44	-
MdBBX20	5.76	40.53	-0.602	A(9.7%)、S(8.4%)、E(7.1%)	62.02	-
MdBBX21	4.37	60.15	-0.906	D(14.8%)、S(12.6%)、A(8.3%)	50.13	-
MdBBX22	4.11	53.65	-0.837	D(15.7%)、S(11.9%)、G(7.0%)	50.65	+
MdBBX23	5.70	46.68	-0.494	S(12.5%)、G(7.5%)、L(7.5%)	61.43	-
MdBBX24	5.90	54.05	-0.651	S(13.2%)、A(9.1%)、G(7.0%)	54.89	+

MdBBX25	6.68	63.24	-0.044	A(12.9%)、C(12.9%)、S(9.7%)	75.65	-
MdBBX26	5.28	45.97	-0.221	S(10.6%)、G(9.4%)、A(7.9%)	68.18	+
MdBBX27	6.44	61.80	-0.503	S(16.8%)、L(8.7%)、E/ K(6.7%)	64.76	+
MdBBX28	5.59	42.24	-0.460	A(9.2%)、S(9.2%)、D(8.1%)	73.87	-
MdBBX29	7.52	48.65	-0.617	S(9.4%)、G(8.5%)、E(7.6%)	56.89	-
MdBBX30	6.59	50.47	-0.485	S(11.9%)、A(7.6%)、P(7.3%)	59.14	-
MdBBX31	6.48	60.25	-0.652	T(11.1%)、L(9.5%)、A(9.5%)	71.27	+
MdBBX32	5.22	49.84	-0.816	D(10.6%)、A(9.3%)、S(8.6%)	56.59	-
MdBBX33	5.22	49.81	-0.816	D(10.6%)、A(9.3%)、S(8.6%)	56.59	+
MdBBX34	9.77	52.59	-0.457	A(16.7%)、K(11.9%)、S(10.3%)	75.26	-
MdBBX35	8.92	57.56	-0.638	L(11.4%)、S(9.7%)、G(9.2%)	70.16	-
MdBBX36	5.71	52.51	-0.230	S(11.6%)、A(8.0%)、G(7.3%)	67.08	+
MdBBX37	5.94	41.22	-0.440	L(10.8%)、S(9.9%)、G(8.8%)	80.34	+
MdBBX38	5.58	44.52	-0.402	L(11.2%)、G(9.6%)、S(9.3%)	76.19	+
MdBBX39	5.56	46.47	-0.544	G(8.3%)、V(8.3%)、A(7.1%)	71.90	+
MdBBX40	8.72	55.70	-0.280	S(14.0%)、R(9.2%)、A(8.9%)	65.87	+
MdBBX41	4.77	57.32	-0.612	E(10.3%)、L(7.8%)、D(7.2%)	69.23	-
MdBBX42	5.65	57.84	-0.383	L(10.4%)、S(9.6%)、E /A(6.9%)	82.03	-
MdBBX43	6.03	43.45	-0.738	S(8.7%)、G(8.6%)、D(8.6%)	59.91	-
MdBBX44	5.33	44.40	-0.808	D(10.6%)、S(9.1%)、G(8.2%)	58.59	+
MdBBX45	6.07	47.10	-0.268	S(10.9%)、A(10.1%)、P(7.7%)	72.53	+
MdBBX46	5.40	36.26	-0.611`	A(10.4%)、S(8.1%)、E(6.9%)	61.88	-
MdBBX47	5.40	36.26	-0.611`	A(10.4%)、S(8.1%)、E(6.9%)	61.88	-
MdBBX48	6.50	26.90	-0.760	S(8.0%)、A(8.0%)、Q(7.6%)	57.45	-
MdBBX49	4.90	64.29	-0.901	S(13.4%)、D(11.5%)、R(9.6%)	55.03	-
MDBBX50	4.53	73.41	-0.997	S(13.6%)、D(13.2%)、R(9.5%)	49.63	-
MdBBX51	6.15	56.20	-0.625	S(14.4%)、A(9.0%)、G(6.8%)	56.36	-
MdBBX52	8.80	52.51	-0.297	S(13.3%)、A(10.4%)、R(9.3%)	66.89	-
MdBBX53	4.58	37.86	-0.320	S(11.5%)、A(8.5%)、L(8.5%)	73.70	-
MdBBX54	5.88	52.74	-0.352	L(10.9%)、R(7.9%)、D(7.4%)	89.31	-
MdBBX55	4.95	40.12	-0.328	S(10.3%)、A(9.5%)、L(8.6%)	76.34	+
MdBBX56	4.79	46.27	-0.150	S(12.8%)、L(10.2%)、A(8.6%)	84.55	+
MdBBX57	3.33	58.69	-0.276	L(12.5%)、S(11.4%)、P(8.3%)	83.49	+
MdBBX58	9.91	37.04	-0.133	A(10.5%)、L(8.8%)、S(7.9%)	85.47	-
MdBBX59	5.00	44.38	-0.307	S(10.7%)、L(10.0%)、A(9.0%)	81.30	+
MdBBX60	7.16	54.70	-0.710	S(11.1%)、L(7.5%)、R(6.9%)	62.88	-
MdBBX61	4.58	69.35	-0.791	S(13.2%)、D(9.7%)、E(9.7%)	56.11	+
MdBBX62	5.59	42.24	-0.460	A(9.2%)、S(9.2%)、V(9.2%)	73.87	+
MdBBX63	5.59	42.24	-0.460	A(9.2%)、S(9.2%)、V(9.2%)	73.87	+
MdBBX64	6.45	48.28	-0.209	S(10.8%)、A(9.9%)、L(8.6%)	76.18	+
MdBBX65	6.68	63.56	-0.015	A(14.5%)、C(12.9%)、S(9.7%)	77.26	-
MdBBX66	5.91	40.69	0.168	A(14.5%)、C(12.8%)、L(12.8%)	95.26	-
MdBBX67	4.61	40.93	-0.465	G(18.2%)、E(13.2%)、V(10.7%)	61.98	+
PcBBX1	5.23	47.38	-0.782	D (10.4), A (7.7), G (8.2)	60.88	+
PcBBX2	5.42	58.32	-0.44	S (13.5), L (9.3), D (6.9)	70.99	-
PcBBX3	5.96	46.7	-0.538	A (10.9), S (10.0), D (7.6)	67.18	+
PcBBX4	4.7	72.38	-0.785	S (13.5), E (9.9), D (8.5)	57.3	-
PcBBX5	8.85	51.52	-0.327	A (10.4), S (13.0), R (10.0)	66.52	+

PcBBX6	6.85	50.96	-0.465	S (11.6), A (8.9), P (7.0)	59.5	-
PcBBX7	4.33	55.09	-0.931	E(14.3), S (11.3), D (10.7)	49.88	-
PcBBX8	5.93	53.77	-0.64	G (9.3), S (9.8), E (7.8)	54.7	+
PcBBX9	5.42	36.04	-0.305	L (12.6), N (8.1), S (9.3)	88.74	-
PcBBX10	5.83	40.32	-0.619	A (9.3), E (7.0), V (6.5)	62.54	-
PcBBX11	4.81	33.55	-0.106	G (13.7), A (9.9), S (9.2)	68.47	+
PcBBX12	5.28	55.89	-0.31	S(12.1), A (7.9), V (6.6)	64.59	-
PcBBX13	6.04	44.86	-0.517	L (9.0), D (8.0), N (7.5)	80	-
PcBBX14	6.41	55.99	-0.707	S (11.7), L (7.8), E (7.0)	64.66	-
PcBBX15	4.58	74.24	-0.931	D (13.3), R (9.3), A (7.7)	50.77	+
PcBBX16	6.08	52.45	-0.608	S(14.2), A (8.7), L (7.0)	59.52	-
PcBBX17	5.82	42.58	-0.573	A (10.6), S (9.2), E (6.7)	63.3	+
PcBBX18	5.70	44.91	-0.518	G (7.4), S (12.0), L (7.4)	59.43	-
PcBBX19	4.08	83.51	-1.19	E(12.9), S (13.6), D (12.9)	42.99	-
PcBBX20	5.93	55.99	-0.408	S (10.6), A (9.6), L (7.0)	63.62	+
PcBBX21	6.66	51.01	-0.377	A (9.9), R (7.7), L (7.7)	77.79	-
PcBBX22	5.25	46.84	-0.074	S 99.6), A (8.5), V (8.1)	76.88	+
PcBBX23	5.91	59.53	-0.617	S (13.7), A (8.2), G (7.0)	53.88	+
PcBBX24	4.94	45.74	-0.242	L (11.1), A (9.1), S (9.4)	85.79	-
PcBBX25	5.61	48.54	-0.518	S (10.3), G (9.8), L (8.5)	67.39	-
PcBBX26	9.14	57.46	-0.314	S (13.3), R (9.6), A (9.2)	66.61	-
PcBBX27	4.55	67.97	-0.933	D (13.4), S (13.0), G (7.7)	49.63	+
PcBBX28	4.36	67.71	-0.112	E(16.5), S 911.2), D (11.2)	48.24	-
PcBBX29	6.51	50.96	-0.367	S (11.5), A (10.0), P (8.0)	69.54	+
PcBBX30	6.1	43.22	0.006	S (11.2), A (7.5), L (9.6)	85.29	-
PcBBX31	5.95	43.84	-0.451	S (10.6), A (10.6), R (7.1)	72.29	-
PcBBX32	5.19	42.18	-0.338	A (10.2), E (8.8), L (7.8)	72.88	-
PcBBX33	5.21	45.91	-0.686	E (9.0), A (8.1), D (8.1)	68.84	+
PcBBX34	5.2	42.96	-0.123	S (11.0), A (10.4), L (8.9)	80.18	+
PcBBX35	5.32	56.21	-0.596	A (9.1), E (9.3), G (7.9)	70.16	+
PcBBX36	5.97	29.57	-0.352	A (8.0), S (8.4), L (7.7)	72.58	-
PcBBX37	5.5	46.64	-0.797	D (10.4), A (8.8), G (7.5)	57.2	+
PcBBX38	6.06	46.05	-0.36	V (10.1), L (8.5), N (8.0)	86.63	+
PcBBX39	5.09	44.18	-0.383	S (11.6), A (9.5), L (7.9)	71.82	-
PcBBX40	4.87	48.94	-0.352	S (11.4), N (9.4), G (8.5)	67.33	+
RcBBX1	5.17	54.22	-0.471	A (10.1), S (13.9), G (7.2)	60.91	+
RcBBX2	6.24	45.62	-0.302	S (11.5), A (10.3), V (7.3)	72.79	+
RcBBX3	4.79	43.02	-0.29	S (10.9), A (9.6), D (7.9)	79.25	-
RcBBX4	6.87	51.57	-0.584	A(10.2), R (8.0), D (8.0)	68.41	+
RcBBX5	4.85	47.63	0.125	G (11.7), S (10.3), A (8.0)	80.09	+
RcBBX6	6.64	41.04	-0.237	S (11.6), L (8.6), A (6.5)	79.01	+
RcBBX7	9.57	56.47	-0.02	S (14.8), R (9.1), G (8.7)	76.06	+
RcBBX8	5.51	57.79	-0.562	S (13.1), L (7.6), G (6.7)	61.13	+
RcBBX9	8.70	32.57	0.038	L (12.2), C (11.2), R (9.2)	92.45	-
RcBBX10	4.83	60.87	-0.924	D (13.7), S (10.2), R (11.0)	58.98	+
RcBBX11	8.59	36.62	-0.459	A (10.5), S (9.2), R (7.8)	63.78	+
RcBBX12	5.52	61.53	-0.674	S (13.0), D (9.1), L (7.1)	59.62	-
RcBBX13	6.36	35.32	0.222	S (11.5), L (11.5), H (7.3)	100.0	-
RcBBX14	7.01	53.61	-0.481	S (13.3), L (8.1), A (6.6)	75.88	-
RcBBX15	5.78	54.12	-0.767	S (11.4), E (8.3), A (8.1)	58	-
RcBBX16	4.91	61.87	-0.313	S (11.6), A (9.1), G (8.8)	60.95	+
RcBBX17	4.31	57.12	-0.815	E (14.1), S (10.8), D (9.5)	58.22	-
RcBBX18	5.98	50.53	-0.47	S (13.0), D (8.3), C (7.4)	65.93	+
RcBBX19	5.79	62.41	-0.671	S (11.3), E (8.0), L (7.7)	58.43	-

RcBBX20	6.09	44.85	-0.628	S (10.5), A (9.0), R (7.7)	67.41	+
RcBBX21	6.11	55.25	-0.523	S (14.1), A (6.7), N (6.7)	62.76	+
RcBBX22	5.74	48.36	-0.686	E (8.8), G (7.8), L (7.6)	66.71	+
PaBBX1	4.77	40.44	-0.315	A (10.9), E (8.4), L (7.9)	74.81	+
PaBBX2	4.95	51.92	-0.046	S (13.2) A (10.8), D (7.5)	82.97	+
PaBBX3	6.80	43.03	-0.458	L (10.5) G (9.5), D (7.2)	74.68	-
PaBBX4	5.15	55.27	-0.79	A (8.7), E (8.4), D (8.7)	56.84	+
PaBBX5	6.88	52.15	-0.818	S (10.8), L (8.8), Q (7.7)	59.43	+
PaBBX6	5.47	46.48	-0.539	S (10.7), A (9.3) V (9.3)	65.95	+
PaBBX7	5.29	48.01	-0.758	G (9.9), S (9.1), R (7.8)	62.06	-
PaBBX8	9.32	53.95	-0.246	S (13.6) A (11.4), G (8.3)	72.16	+
PaBBX9	5.13	41.15	-0.426	A (8.9), L (9.8), S (9.8)	72.37	+
PaBBX10	5.52	56.34	-0.508	S (13.2), A (8.9), L (6.9)	60.02	-
PaBBX11	5.07	57.97	-0.485	S (13.8), G (8.5), N (7.5)	63.13	-
PaBBX12	9.13	59.62	-0.161	C (10.3), L (11.1), R (11.1)	77.44	-
PaBBX13	9.09	88.73	-0.849	R (12.5), S (13.2), A (9.2)	60.46	+
PaBBX14	5.78	43.12	-0.482	A (11.4), S (9.3), E (7.1)	67.25	-
PaBBX15	4.51	60.61	-1.254	D (12.3), S (12.3), E (10.3)	38.58	+
PaBBX16	5.53	63.35	-0.327	S (12.7), G (10.7), A (8.3)	63.03	+
PaBBX17	4.78	58.79	-0.698	D (10.9), R (8.9), E (9.9)	73.7	+
PaBBX18	5.12	48.46	-0.77	D (8.5), E (8.5), A (8.1)	62.23	-
PaBBX19	8.51	48.36	-0.387	A (9.2), R (8.3), S (7.0)	74.1	+
PaBBX20	8.71	56.35	-0.329	L (9.3), R (7.7), S (7.7)	83.41	+
PaBBX21	5.97	58.23	-0.462	S (13.9), A (7.2), C (6.7)	58.17	+
PaBBX22	6.91	51.57	-0.661	S (11.8), A (7.2), G (7.4)	58.9	-
RoBBX1	6.59	51.14	-0.404	(L)8.4, (R)7.6, (A)7.2	75.58	-
RoBBX2	6.14	46.38	-0.502	(N)8.7, (V)8.2, (L)7.2	73.99	-
RoBBX3	5.69	44.14	-0.461	(S)11.6, (D)8.8, (L)7.4	68.47	+
RoBBX4	5.69	42.44	-0.448	(S)10.6, (A)8.6, (D)8.0	71.33	-
RoBBX5	5.97	53.09	-0.331	(S)13.6, (A)7.3, (D)7.0	69.37	-
RoBBX6	5.33	56.75	-0.76	(S)11.6, (L)8.1, (G)7.3	54.63	+
RoBBX7	4.39	58.98	-0.936	(E)13.0, (S)10.5, (D)10.1	58.79	+
RoBBX8	5.17	59.8	-0.311	(S)12.8, (A)9.2, (G)7.8	59.86	-
RoBBX9	4.46	75.87	-1.046	(S)12.3, (E)11.8, (D)8.8	44.87	+
RoBBX10	5.46	54.7	-0.724	(S)10.6, (D)9.2, (A)9.0	59.84	+
RoBBX11	6.17	39.79	-0.663	(G)9.0, (L)7.9, (N)7.3	64.55	+
RoBBX12	6.50	47.22	-0.308	(S)11.0, (A)8.8, (P)8.2	71.34	-
RoBBX13	5.11	49.66	-0.746	(E)10.8, (G)8.4, (L)8.2	63.11	+
RoBBX14	8.43	53.94	-0.377	(S)12.5, (A)8.8, (R)8.3	77.22	+
RoBBX15	5.39	35.9	-0.588	(A)9.8, (S)8.8, (D)7.3	60.91	+
RoBBX16	4.58	58.52	-0.927	(D)14.0, (S)11.7, (R)9.4	56.04	-
RoBBX17	6.69	49.97	-0.093	(L)12.9, (C)9.5, (E)7.8	86.55	-
RoBBX18	5.26	55.72	-0.484	(S)12.6, (L)8.2, (G)7.1	67.17	+
RoBBX19	7.95	40.25	-0.086	(S)11.0, (L)8.2, (A)7.7	78.24	-
RoBBX20	9.17	58.61	-0.155	(S)12.9, (A)10.5, (R)9.4	72.07	+
PpBBX1	5.94	47.97	-0.304	(S)11.7, (A)10.7, (D)6.5	73.92	
PpBBX2	4.70	43.55	-0.279	(A)11.3, (S)9.6, L (8.8)	78.49	+
PpBBX3	5.53	48.83	-0.651	(G)11.2, (L)8.9, (D)7.7	63.67	-
PpBBX4	5.07	55.85	-0.801	(E)9.2, (S)8.5, (G)8.3	53.27	+
PpBBX5	5.78	64.19	-0.468	(S)13.5, (L)9.8 (D) 7.6	68.48	+
PpBBX6	7.56	45.64	-0.287	(S)12.1, (T)8.7, (A) 8.2	72.61	+
PpBBX7	5.56	43.24	-0.497	(A)10.8, (S)10.5, (V)9.0	66.56	-
PpBBX8	8.94	46.95	-0.374	(S)16.6, (A)10.7, (C)7.7	54.97	-
PpBBX9	5.06	39.46	-0.38	(S)10.1, (A)8.9, (D)7.7	71.54	+

PpBBX10	5.63	55.89	-0.485	(S)12.9, (A)9.6, (G)7.4	60.74	+
PpBBX11	5.74	53.96	-0.556	(S)12.2, (G)8.4, (N)7.3	58.4	+
PpBBX12	9.32	59.93	-0.247	(R)12.0, (L)11.1, (C)9.4	77.44	+
PpBBX13	4.73	74.93	-1.067	(S)14.4,(D)11.1, (R)8.5	49.37	-
PpBBX14	5.22	46.66	-0.588	(A)9.8, (S)8.8, (E)7.3	63.99	+
PpBBX15	4.38	63.21	-0.765	(E)13.3, (S)10.8 (D) 10.0	68.29	+
PpBBX16	4.94	47.44	-0.774	(E)9.3, (D)8.9, (L)8.0	64.77	-
PpBBX17	7.11	49.07	-0.704	(R)8.3, (D)7.6, (G)7.0	67.64	
PpBBX18	5.75	59.38	-0.377	(V)9.1, (L)7.7, (A)7.2	78.7	-
PpBBX19	6.64	56.08	-0.471	(S)13.9, (A)7.2, (L)6.7	56.78	+
PpBBX20	6.03	50.38	-0.654	(S)11.3, (L)8.2, (E)7.7	67.64	-
FvBBX1	6.74	51.56	-0.570	(A)10.2, (D)8.6, (R) 8.1	68.23	+
FvBBX2	6.17	47.96	-0.622	(S)13.7, (D)7.5, (A)6.6	58.44	+
FvBBX3	5.34	56.44	-0.506	(S)10.3, (G)8.8, (A)6.1	60.39	-
FvBBX4	6.62	49.21	-0.461	(S)14.1, (T)7.3, (N)6.1	66.36	+
FvBBX5	6.33	48.1	-0.512	(S)11.9, (A)10.9, (D)7.4	69.49	-
FvBBX6	4.37	65.89	-1.086	(S)12.6, (D)12.0,(E)10.9	44.75	+
FvBBX7	5.12	56.32	-0.313	(S)10.8, (G)9.1, (A) 8.7	67.28	+
FvBBX8	4.30	54.21	-0.756	(E)14.6, (D)10.1, (S)8.9	63.52	-
FvBBX9	5.63	55.03	-0.304	(S)12.4, (A)10.4, (P)7.9	68.29	+
FvBBX10	4.71	39.45	-0.318	(S)9.7, (D)8.4, (E)6.7	76.72	-
FvBBX11	6.51	30.39	-0.186	(L)13.4, (S)9.0, (N)8.0	91.95	+
FvBBX12	5.87	56.74	-0.006	(S)13.5, (L)12.3, (A)8.0	88.59	-
FvBBX13	5.62	56.7	-0.654	(S)12.3, (D)8.5, (L)6.8	61.87	-
FvBBX14	5.56	58.44	-0.799	(E)9.1, (A)8.4, (R)8.0	55.56	-
FvBBX15	5.56	48.82	-0.781	(E)9.4, (G)7.9, (D)7.7	62.36	-
FvBBX16	8.99	54.72	-0.161	(S)13.6,(R)10.6, (A)9.8	73.9	-
FvBBX17	5.51	53.63	0.019	(C)12.9,(A)11.4, (L)8.6	79.57	-
FvBBX18	5.45	54.48	-0.502	(S)12.9, (A)9.1, (L)7.4	60.19	+
FvBBX19	5.43	57.31	-0.524	(S)12.2,(L)8.1, (G)7.0	63.04	+
FvBBX20	8.75	54.05	0.008	(L)10.7, (R)9.8, (C)8.9	95.8	-
FvBBX21	4.71	51.57	-0.902	(D)13.9, (S)11.1,(R)9.5	60.08	+

Table S2. **Abbreviations:** Pl, protein length; pIs, isoelectric point; Ii, index instability; Ai, aliphatic index; GRAVY, grand average of hydropathicity; (A, Ala; P, Pro; S, Ser; G, Gly; L, Leu:N, ASN; T,Thr).The information about protein was taken from ExPASy (http://web.expasy.org/protparam/)

Figure S1a: Detail alignment sequences of B-BOX1 domain of BBX gene family of seven *Rosacea* species.

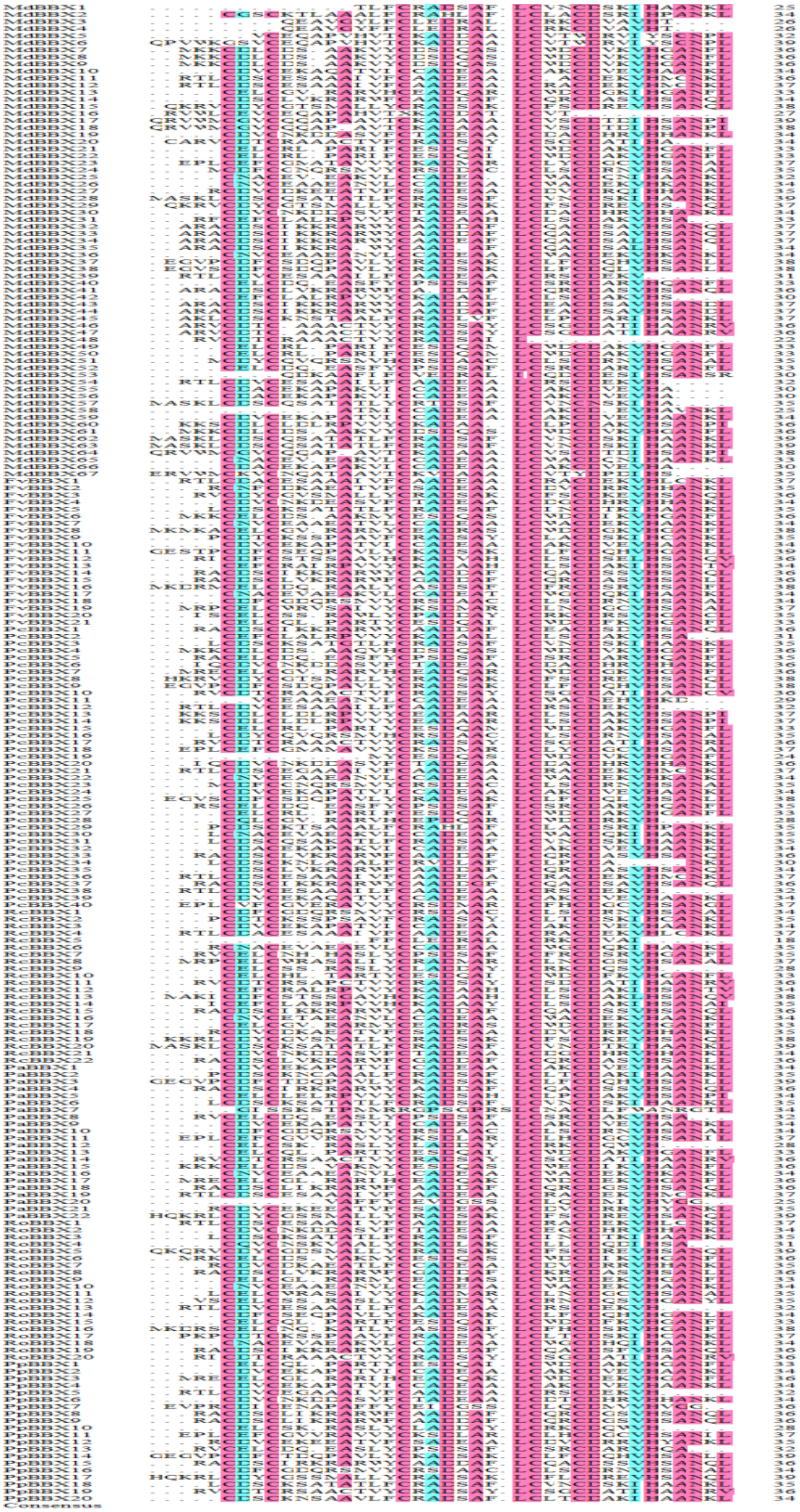

Figure S1b: Detail alignment sequences of B-BOX2 domain of BBX gene family of seven *Rosacea* species.

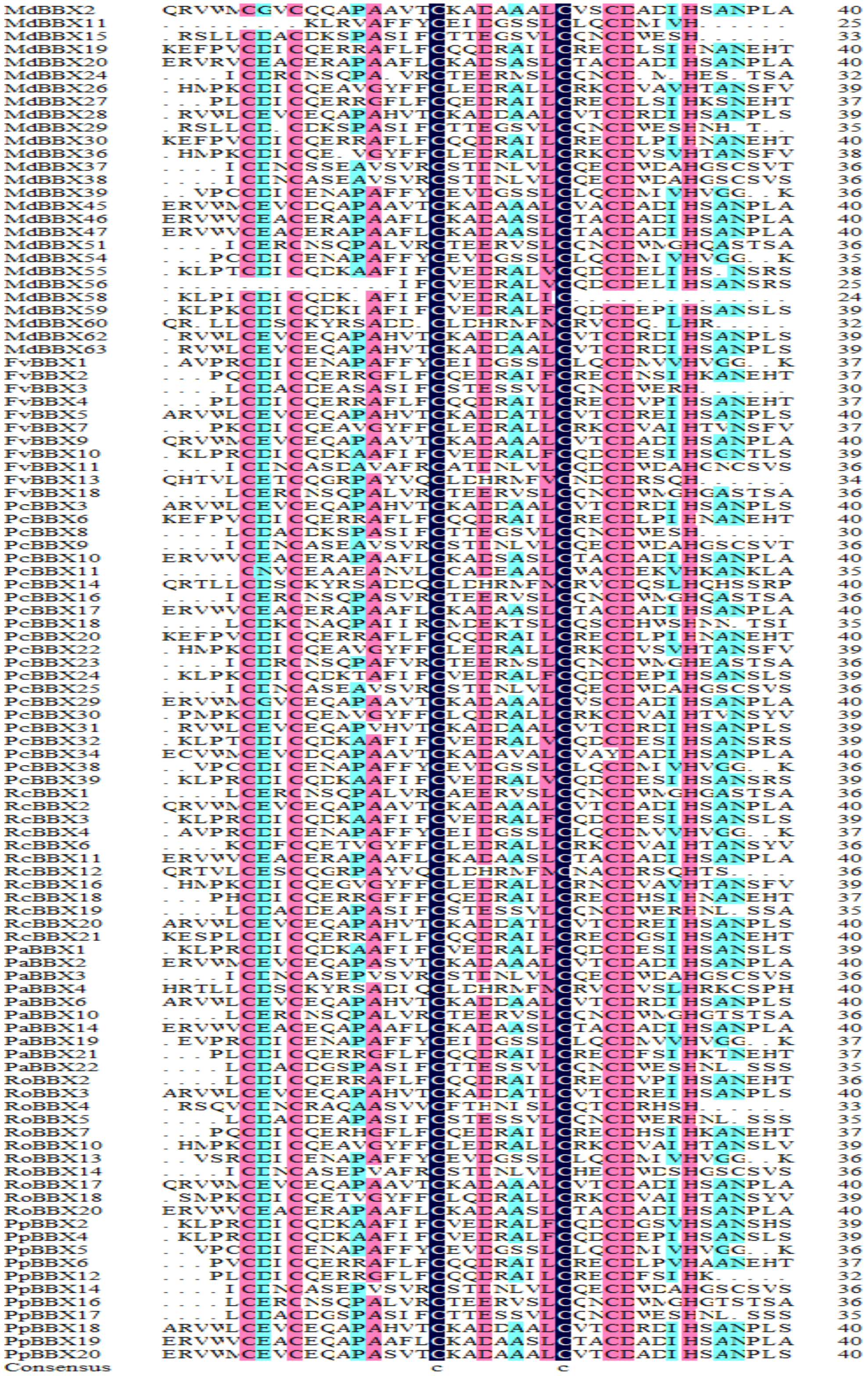

Figure S1c: Detail alignment sequences of CCT domain of BBX gene family of seven *Rosacea* species.

```
MdBBX1      ..REARVLRYREKRKNRKFEKTIRYASRKAYAETRPRIKG   38
MdBBX2      ..................FQKTIRYASRKAYAETRPRIKG   22
MdBBX14     ..REARVSRYREKRRTRLFSKKIRYEVRKLNAEKRPRMKG   38
MdBBX20     ..REARVLRYREKKKTRKFEKTIRYASRKAYAETRPRIKG   38
MdBBX23     ..RDKAKMRYKEKKKTRTFGKQIRYASRKARADTRKRVKG   38
MdBBX24     ..RSNAVMRYKEKKKARKFEKRVRYASRKARADVRKRVKG   38
MdBBX29     ..RETALSRYKEKKKTRRYEKHIRYESRKVRAESRTRIKG   38
MdBBX32     ..REARVSRYREKRRTRLFSKKIRYEVRKLNAEKRPRMKG   38
MdBBX33     ..REARVSRYREKRRTRLFSKKIRYEVRKLNAEKRPRMKG   38
MdBBX37     ..RCNAMLRYKEKKKTRRYDKHIRYESRKARADTRKRVKG   38
MdBBX38     ..RCNAMLRYKEKKKTRRYEKHIRYESRKARADTRMRVKG   38
MdBBX43     ..REARVSRYREKRRTRLFSKKIRYEVRKLNAEKRPRMKG   38
MdBBX44     ..REARVSRYREKRRTRLFSKKIRYEVRKLNAEKRPRMKG   38
MdBBX45     ..REARVLRYREKRKNRKFQKTIRYASRKAYAETRPRIKG   38
MdBBX46     ..REARVLRYREKKKTRKFEKTIRYASRKAYAETRPRIKG   38
MdBBX47     ..REARVLRYREKKKTRKFEKTIRYASRKAYAETRPRIKG   38
MdBBX48     ..REARVLRYREKKKTRKFEKTIRYASRKAYAETRPRIKG   38
MdBBX51     ..RSNAVMRYKEKKKARKFEKRVRYASRKARADVRKRVKG   38
FvBBX3      ..RESALTRYKEKKKTRRYCNLIMSGSRRFV.........   29
FvBBX5_     ..REARVLRYREKRKNRKFEKTIRYASRKAYAETRPRIKG   38
FvBBX11_    ..RCNAMLRYKEKKKNRRYDKHIRYESRKARADTRKRVKG   38
FvBBX13_    ..RANSLMRYKEKKQSRLTEKKFPYSPRKATVDVHRRGRG   38
FvBBX14_    ..REARVSRYREKRRTRLFSKKIRYEVRKLNAEKRPRMKG   38
FvBBX15_    ..REARVSRYREKRRTRLFSKKIRYEVRKLNAEKRPRMKG   38
FvBBX18_    ..RSNAVMRYKEKKKARKFEKRVRYASRKARADVRKRVKG   38
FvBBX19_    ..RDKAKMRYEEKKKTRTFGKQIRYASRKARADTRKRVKG   38
PcBBX1_     ..REARVSRYREKRRTRLFSKKIRYEVRKLNAEKRPRMKG   38
PcBBX3_     ..REARVLRYREKRKNRKFEKTIRYASRKAYAETRPRIKG   38
PcBBX8_     ..RETALSRYKEKKKTRRYEKHIRYESRKVRAESRTRIKG   38
PcBBX9_     ..RCNAMLRYKEKKKTRRYDKHIRYESRKARADTRKRVKG   38
PcBBX10_    ..REARVLRYREKKKTRKFEKTIRYASRKAYAETRPRIKG   38
PcBBX16_    ..RSNAVMRYKEKKKARKFEKRVRYASRKARADVRKRVKG   38
PcBBX17_    ..REARVLRYREKKKTRKFEKTIRYASRKAYAETRPRIKG   38
PcBBX18_    ..RDKAKMRYKEKKKTRTFGKQIRYASRKARADTRKRVKG   38
PcBBX23     ..RSNAVMRYKEKKKARKFEKRVRYASRKARADVRKRVKG   38
PcBBX25     ..RCNAMLRYKEKKKTRRYEKHIRYESRKARADTRKRVKG   38
PcBBX29     ..REARVLRYREKRKNRKFQKTIRYASRKAYAETRPRIKG   38
PcBBX30     ..RVARVLRYREKRKNRKFEKTIRYASRKAYAETRPRIKG   38
PcBBX33     ..REARVSRYREKRRTRLFSKKIRYEVRKLNAEKRPRMKG   38
PcBBX35     ..REARVSRYREKRRTRLFSKKIRYEVRKLNAEKRPRMKG   38
PcBBX37     ..REARVSRYREKRRTRLFSKKIRYEVRKLNAEKRPRMKG   38
RcBBX1      ..RSNAVMRYKEKKKARKFEKRVRYASRKARADVRKRVKG   38
RcBBX2      ..REARVMRYREKRKNRKFQKTIRYASRKAYAETRPRIKG   38
RcBBX8      ..RDKAKMRYEEKKKTRTFGKQIRYASRKARADTRKRVKG   38
RcBBX11     ..REARVLRYREKKKTRKFEKTIRYASRKAYAETRPRIKG   38
RcBBX12     .......MRYKEKKHSRLTEKKLPYPSRKATTNIHKLGKG   33
RcBBX14     ..RAKAMIRYKEKKRSRSHAKTIRYPNRKARAAVRKRVNG   38
RcBBX15     ..REARVSRYREKRRTRLFSKKIRYEVRKLNAEKRPRMKG   38
RcBBX19     ..REARVLRYREKRKNRKFEKTIRYASRKAYAETRPRIKG   38
RcBBX22     ..REARVSRYREKRRTRLFSKKIRYEVRKLNAEKRPRMKG   38
PaBBX3      ..RCNAMLRYKEKKKTRRYDKHIRYESRKARADTRKRIKG   38
PaBBX4      ..REARVSRYREKRRTRLFSKKIRYEVRKLNAEKRPRMKG   38
PaBBX6      ..REARVLRYREKRKNRKFEKTIRYASRKAYAETRPRIKG   38
PaBBX7      ..RIASLVRFREKRKERCFDKKIRYTVRKEVAQSLRR...   35
PaBBX10     ..RSNAVMRYKEKKKARKFDKRVRYASRKARADVRKRVKG   38
PaBBX11     ..RDKAKMRYKEKKKTRTY.....................   17
PaBBX14     ..REARVLRYREKKKTRKFEKTIRYASRKAYAETRPRIKG   38
PaBBX15     HMPKCDICQEAVGYFFCLEDRALLCRTCDVAVHAANSLVS   40
PaBBX18     ..REARVSRYREKRRTRLFSKKIRYEVRKLNAEKRPRMKG   38
PaBBX22     ..RETALSRYKAKKKTRRYEKHIRYESRKVRAESRTRIKG   38
RoBBX3      ..REARVLRYREKRKNRKFEKTIRYASRKAYAETRPRIKG   38
RoBBX5      ..RESALSRYKEKKKTRRYDKHIRYESRKVRAESRTRIKG   38
RoBBX8      ..REARVSRYREKRRTRLFSKKIRYEVRKLNAEKRPRMKG   38
RoBBX11     ..RDKAKMRYEEKKKTRTFGKQIRYASRKARADTRKRVKG   38
RoBBX14     ..RCNAMLRYKEKKKNRRYDKHIRYESRKARADTRKRVKG   38
RoBBX17     ..REARVMRYREKRKNRKFQKTIRYASRKAYAETRPRIKG   38
RoBBX19     ..REARVSRYKEKRRTRLFSKKIRYEVRKLNAEKRPRMKG   38
RoBBX20     ..REARVLRYREKKKTRKFEKTIRYASRKAYAETRPRIKG   38
PpBBX8      ..REARVSRYREKRRTRLFSKKIRYEVRKLNAEKRPRMKG   38
PpBBX9      ..REARVSRYREKRRTRLFSKKIRYEVRKLNAEKRPRMKG   38
PpBBX11     ..RDKAKMRYKEKKKTRMFGKQIRYASRKARADTRKRVKG   38
PpBBX14     ..RCNAMLRYKEKKKTRRYDKHIRYESRKARADTRKRVKG   38
PpBBX15     ..REARVSRYREKRRTRLFSKKIRYEVRKLNAEKRPRMKG   38
PpBBX16     ..RSNAVMRYKEKKKARKFDKRVRYASRKARADVRKRVKG   38
PpBBX17     ..RETALSRYKEKKKTRRYEKHIRYESRKVRAESRTRIKG   38
PpBBX18     ..REARVLRYREKRKNRKFEKTIRYASRKAYAETRPRIKG   38
PpBBX19     ..REARVLRYREKKKTRKFEKTIRYASRKAYAETRPRIKG   38
PpBBX20     ..REARVLRYREKRKNRKFQKTIRYASRKAYAETRPRIKG   38
Consensus
```

Figure S2: Structures of the BBX proteins. The length and order of the domains represent their actual location within each protein.

Name	Domains	BBX1	BBX2	CCT	Protein structure
**MdBBX1**	1BBX+CCT	3-36		252-293	
**MdBBX2**	2BBX+CCT	19-61	67-104	350-377	
**MdBBX3**	1BBX	51-76			
**MdBBX4**	1BBX	10,35			
**MdBBX5**	1BBX	57-97			
**MdBBX6**	1BBX	139-186			
**MdBBX7**	1BBX	1-45			
**MdBBX8**	1BBX	1-45			
**MdBBX9**	1BBX	1-45			
**MdBBX10**	1BBX	5-47			
**MdBBX11**	2BBX	2-45	84-108		
**MdBBX12**	1BBX	2-45			
**MdBBX13**	1BBX	4-45			
**MdBBX14**	1BBX+CCT	23-65		377-419	
**MdBBX15**	2BBX	17-63	70-92		
**MdBBX16**	1BBX	28-54			
**MdBBX17**	1BBX	19-66			
**MdBBX18**	1BBX	19-66			
**MdBBX19**	2BBX	5-47	53-100		
**MdBBX20**	2BBX+CCT	16-49	58-105	323-365	
**MdBBX21**	1BBX	4-45			
**MdBBX22**	1BBX	4-45			
**MdBBX23**	1BBX+CCT	2-47		400-443	
**MdBBX24**	2BBX+CCT	4-47	47-90	356-399	
**MdBBX25**	1BBX	5-47			
**MdBBX26**	2BBX	5-47	51-97		
**MdBBX27**	2BBX	4-47	56-100		
**MdBBX28**	2BBX	1-48	45-91		
**MdBBX29**	2BBX+CCT	14-60	57-92	287-330	
**MdBBX30**	2BBX	5-47	53-100		
**MdBBX31**	1BBX	3-35			
**MdBBX32**	1BBX+CCT	16-61		399-441	
**MdBBX33**	1BBX+CCT	16-61		399-441	
**MdBBX34**	1BBX	16-61			
**MdBBX35**	1BBX	16-61			

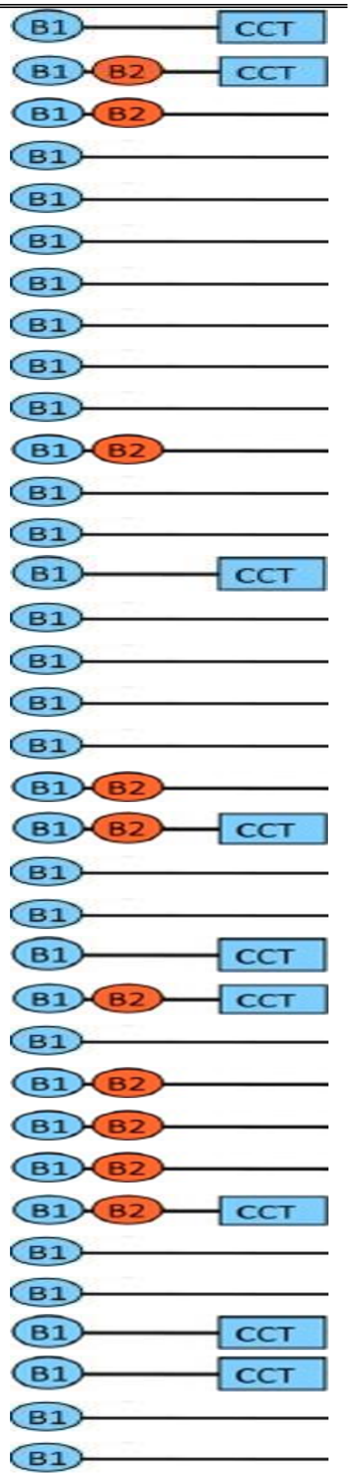

**MdBBX36**	2BBX	5-47	51-97	
**MdBBX37**	2BBX+CCT	10-56	56-99	335-377
**MdBBX38**	2BBX+CCT	10-56	56-99	436-505
**MdBBX39**	2BBX	26-56	97-140	
**MdBBX40**	1BBX	8-49		
**MdBBX41**	1BBX	429-474		
**MdBBX42**	1BBX	278-307		
**MdBBX43**	1BBX+CCT	146-191		528-571
**MdBBX44**	1BBX+CCT	16-61		398-441
**MdBBX45**	2BBX+CCT	59-104	110-147	394-436
**MdBBX46**	2BBX+CCT	17-62	68-105	324-366
**MdBBX47**	2BBX+CCT	17-62	68-105	324-366
**MdBBX48**	1BBX+CCT	18-39		206-248
**MdBBX49**	1BBX	76-117		
**MDBBX50**	1BBX	4-45		
**MdBBX51**	2BBX+CCT	4-47	47-82	90-93
**MdBBX52**	1BBX	8-49		
**MdBBX53**	1BBX	21-58		
**MdBBX54**	2BBX	2-33	54-96	
**MdBBX55**	2BBX	5-34	55-100	
**MdBBX56**	2BBX	5-34	91-122	
**MdBBX57**	1BBX	1-35		
**MdBBX58**	2BBX	287-321	329-353	
**MdBBX59**	2BBX	5-47	53-98	
**MdBBX60**	2BBX	2-47	49-77	
**MdBBX61**	1BBX	1-45		
**MdBBX62**	2BBX	1-48	45-91	
**MdBBX63**	2BBX	1-48	45-91	
**MdBBX64**	1BBX	19-66		
**MdBBX65**	1BBX	5-47		
**MdBBX66**	1BBX	5-34		
**MdBBX67**	1BBX	13-47		
**PcBBX1**	1BBX+CCT	17-57		397-440
**PcBBX2**	1BBX	3-35		
**PcBBX3**	2BBX+CCT	5-58	44-91	279-321
**PcBBX4**	1BBX	1-45		
**PcBBX5**	1BBX	6-49		
**PcBBX6**	2BBX	3-47	53-100	
**PcBBX7**	1BBX	1-45		
**PcBBX8**	2BBX+CCT	14-60	60-89	343-386
**PcBBX9**	2BBX+CCT	10-56	56-99	439-481

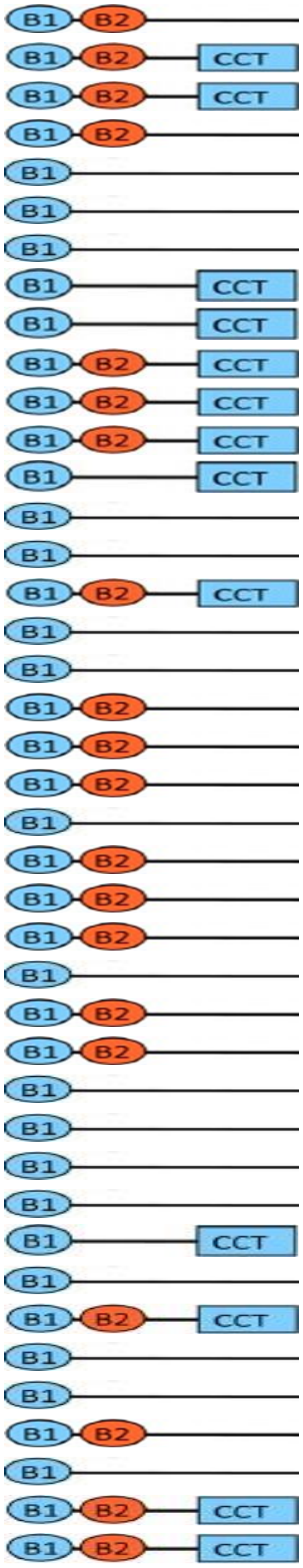

**PcBBX10**	2BBX+CCT	16-52	58-105	309-371
**PcBBX11**	1BBX	7-35	55-97	
**PcBBX12**	1BBX	3-35		
**PcBBX13**	1BBX	2-47		
**PcBBX14**	2BBX	2-40	43-88	
**PcBBX15**	1BBX	4-45		
**PcBBX16**	2BBX+CCT	4-47	47-90	356-399
**PcBBX17**	2BBX+CCT	18-62	58-105	289-331
**PcBBX18**	2BBX+CCT	2-47	47-81	402-445
**PcBBX19**	1BBX	1-31		
**PcBBX20**	2BBX	3-47	53-100	
**PcBBX21**	1BBX	2-45		
**PcBBX22**	2BBX	5-47	51-97	
**PcBBX23**	2BBX+CCT	4-47	47-90	356-399
**PcBBX24**	2BBX	4-45	53-98	
**PcBBX25**	2BBX+CCT	10-56	56-99	434-476
**PcBBX26**	1BBX	6-49		
**PcBBX27**	1BBX	4-45		
**PcBBX28**	1BBX	4-31		
**PcBBX29**	2BBX+CCT	18-61	57-104	335-378
**PcBBX30**	2BBX+CCT	4-47	53-99	279-321
**PcBBX31**	2BBX	5-48	45-91	
**PcBBX32**	2BBX	5-47	55-100	
**PcBBX33**	1BBX+CCT	17-61		362-404
**PcBBX34**	1BBX	18-56	52-99	
**PcBBX35**	1BBX+CCT	19-61		372-421
**PcBBX36**	1BBX	2-45		
**PcBBX37**	1BBX	17-61		
**PcBBX38**	2BBX	2-33	53-99	
**PcBBX39**	2BBX	5-47	55-100	
**PcBBX40**	1BBX	2-47		
**RcBBX1**	2BBX+CCT	5-47	47-90	358-401
**RcBBX2**	2BBX+CCT	16-59	59-102	285-327
**RcBBX3**	2BBX	5-47	53-96	
**RcBBX4**	2BBX	2-45	52-96	
**RcBBX5**	1BBX	46-63		
**RcBBX6**	2BBX	21-62	73-116	
**RcBBX7**	1BBX	4-47		
**RcBBX8**	1BBX+CCT	1-43		394-437
**RcBBX9**	1BBX	21-48		
**RcBBX10**	1BBX	4-45		

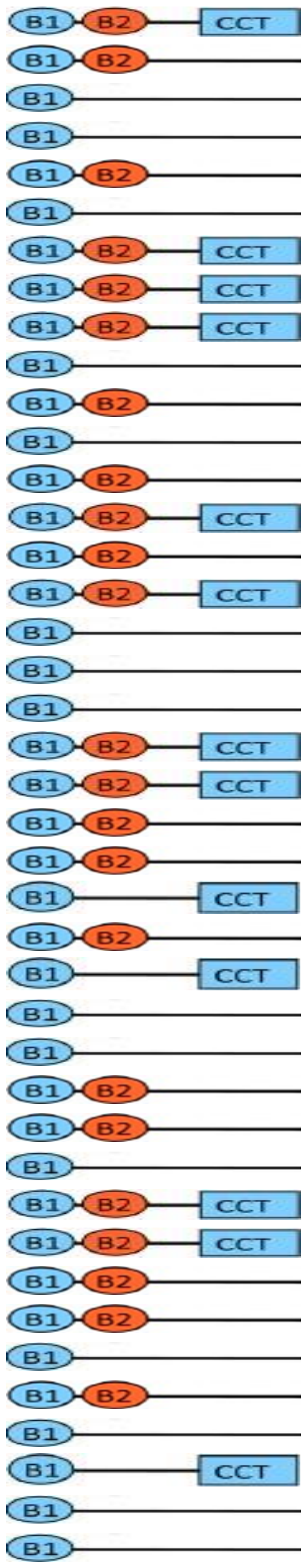

**RcBBX11**	2BBX+CCT	18-62	58-105	225-267
**RcBBX12**	2BBX+CCT	5-39	43-78	401-439
**RcBBX13**	1BBX	1-47		
**RcBBX14**	1BBX+CCT	4-44		307-347
**RcBBX15**	1BBX+CCT	25-69		384-426
**RcBBX16**	2BBX	5-47	51-97	
**RcBBX17**	1BBX	4-45		
**RcBBX18**	2BBX	4-47	56-100	
**RcBBX19**	2BBX+CCT	21-67	67-109	345-386
**RcBBX20**	2BBX	72-119	115-164	
**RcBBX21**	2BBX	5-47	54-100	
**RcBBX22**	1BBX+CCT	71-115		442-484
**PaBBX1**	2BBX	5-47	53-98	
**PaBBX2**	2BBX	19-62	58-100	
**PaBBX3**	2BBX+CCT	9-56	56-99	443-485
**PaBBX4**	2BBX+CCT	17-61	43-90	395-437
**PaBBX5**	1BBX	5-47		
**PaBBX6**	2BBX+CCT	5-72	44-91	282-324
**PaBBX7**	1BBX	202-259		
**PaBBX8**	1BBX	4-35		
**PaBBX9**	1BBX	5-44		
**PaBBX10**	2BBX+CCT	5-47	47-90	407-441
**PaBBX11**	1BBX	2-47		
**PaBBX12**	1BBX	24-51		
**PaBBX13**	1BBX	4-45		
**PaBBX14**	2BBX+CCT	17-61	57-100	255-297
**PaBBX15**	1BBX+CCT	2-46		51-97
**PaBBX16**	1BBX	5-47		
**PaBBX17**	1BBX	1-45		
**PaBBX18**	1BBX+CCT	17-61		140-452
**PaBBX19**	2BBX	2-45	52-96	
**PaBBX20**	1BBX	37-70		
**PaBBX21**	2BBX	4-45	56-100	
**PaBBX22**	2BBX+CCT	19-66	66-108	347-390
**RoBBX1**	1BBX	2-45		
**RoBBX2**	2BBX	5-47	58-100	
**RoBBX3**	2BBX+CCT	5-48	44-91	278-320
**RoBBX4**	1BBX	5-35	44-76	
**RoBBX5**	2BBX+CCT	29-76	76-118	351-394
**RoBBX6**	1BBX	1-45		
**RoBBX7**	2BBX	4-47	56-100	

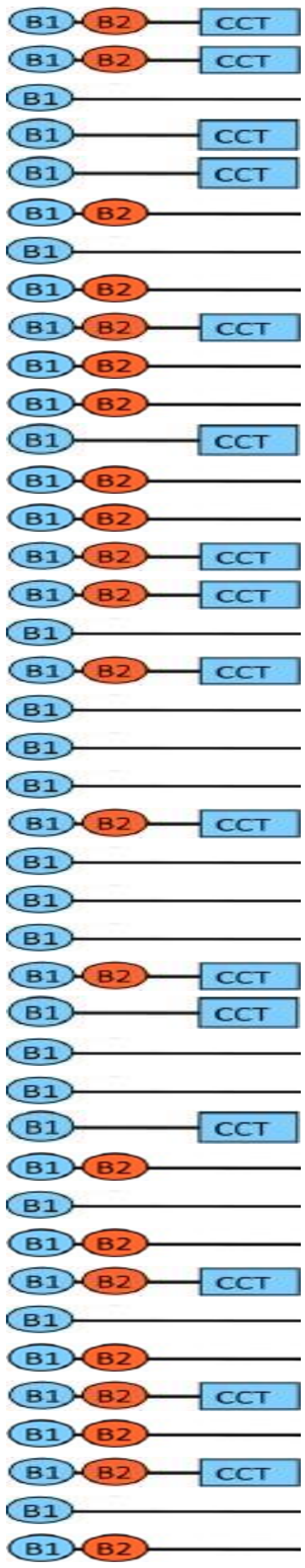

**RoBBX8**	1BBX+CCT	24-68		401-443
**RoBBX9**	1BBX	4-45		
**RoBBX10**	2BBX	5-47	51-97	
**RoBBX11**	1BBX+CCT	4-47		397-440
**RoBBX12**	1BBX	19-62		
**RoBBX13**	2BBX	2-33	53-96	
**RoBBX14**	2BBX+CCT	14-56	56-99	431-473
**RoBBX15**	1BBX	4-45		
**RoBBX16**	1BBX	1-47		
**RoBBX17**	2BBX+CCT	14-59	55100	292-334
**RoBBX18**	2BBX	5-47	52-98	
**RoBBX19**	1BBX+CCT	25-69		387-429
**RoBBX20**	2BBX+CCT	17-61	57-104	327-369
**PpBBX1**	1BBX	35-76		
**PpBBX2**	2BBX	5-47	55-100	
**PpBBX3**	1BBX	1-45		
**PpBBX4**	2BBX	5-47	53-98	
**PpBBX5**	2BBX	2-33	53-96	
**PpBBX6**	2BBX	5-47	56-100	
**PpBBX7**	1BBX	25-69		
**PpBBX8**	1BBX+CCT	17-61		389-431
**PpBBX9**	1BBX+CCT	17-61		389-431
**PpBBX10**	1BBX	24-51		
**PpBBX11**	1BBX+CCT	2-47		395-438
**PpBBX12**	2BBX	4-45	56-87	
**PpBBX13**	1BBX	4-35		
**PpBBX14**	2BBX+CCT	9-56	56-99	445-487
**PpBBX15**	1BBX+CCT	17-61		392-434
**PpBBX16**	2BBX+CCT	5-47	47-90	359-402
**PpBBX17**	2BBX+CCT	19-66	66-108	347-390
**PpBBX18**	2BBX+CCT	5-48	44-91	282-324
**PpBBX19**	2BBX+CCT	17-61	57-104	317-359
**PpBBX20**	2BBX+CCT	20-62	58-105	311-353
**FvBBX1**	2BBX	2-45	52-95	
**FvBBX2**	2BBX	4-47	52-100	
**FvBBX3**	2BBX+CCT	27-75	76-105	349-377
**FvBBX4**	2BBX	5-47	57-100	
**FvBBX5**	2BBX+CCT	8-51	47-94	251-393
**FvBBX6**	1BBX	1-45		
**FvBBX7**	2BBX	5-45	53-97	
**FvBBX8**	1BBX	1-47		

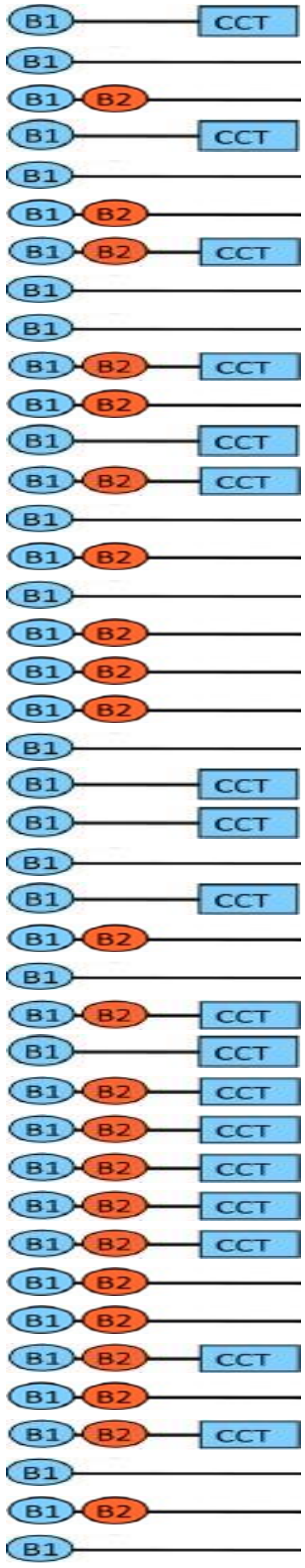

**FvBBX9**	2BBX	15-59	55-102		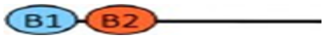
**FvBBX10**	2BBX	5-47	53-98		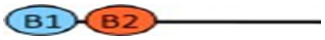
**FvBBX11**	2BBX+CCT	9-56	56-99	430-472	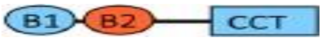
**FvBBX12**	1BBX	3-39			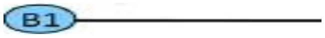
**FvBBX13**	2BBX+CCT	5-39	56-99	427-470	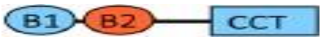
**FvBBX14**	1BBX+CCT	25-69		393-435	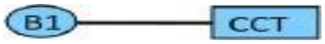
**FvBBX15**	1BBX+CCT	17-61		386-428	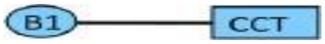
**FvBBX16**	1BBX	1-47			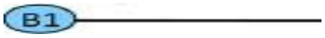
**FvBBX17**	1BBX	5-47			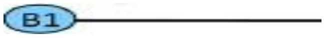
**FvBBX18**	2BBX+CCT	5-47	47-90	358-401	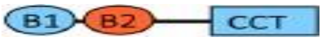
**FvBBX19**	1BBX+CCT	1-45		388-431	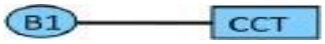
**FvBBX20**	1BBX	2-63			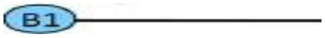
**FvBBX21**	1BBX	4-45			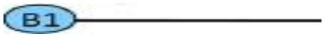

Listed abbreviations: B1=B-box1, B2=B-box2

Figure S3. The exon/intron structures and identified motifs of BBX family genes. The structure of individual *BBX* gene was obtained through the Gene Structure Display Server (http://gsds. cbi.pku.edu.cn) by aligning the coding or cDNA sequences with their corresponding genomic DNA sequence. 10 motifs were identified through online meme tool.

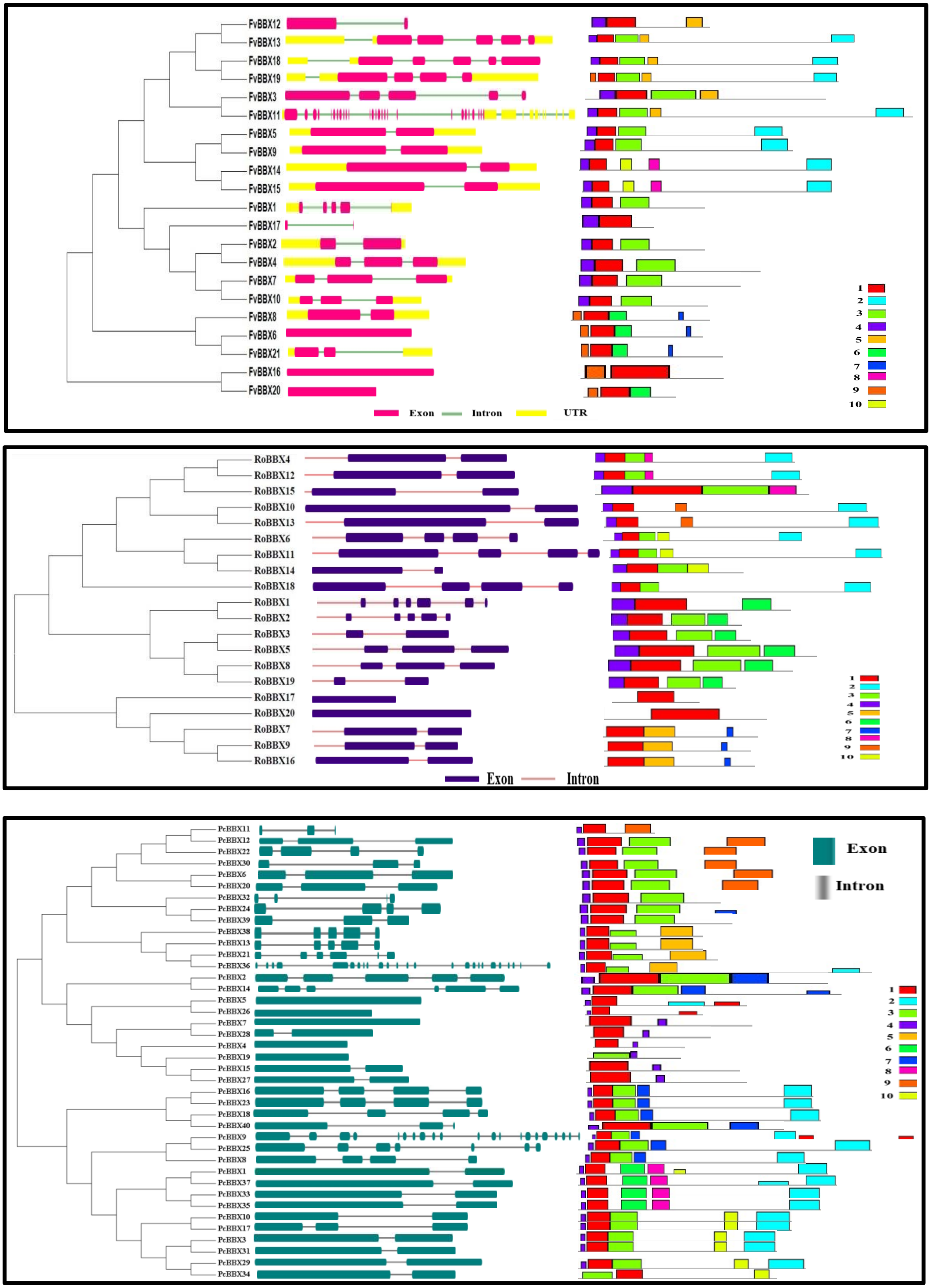

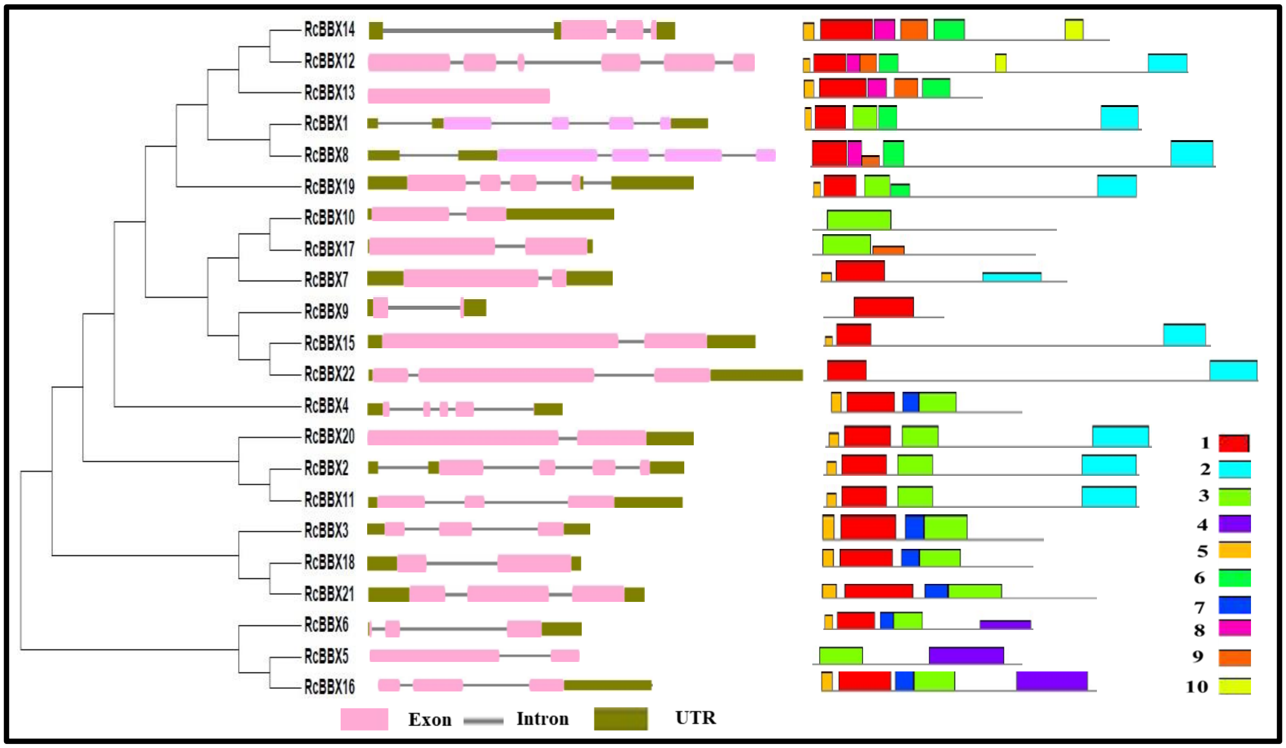
RcBBX14
RcBBX12
RcBBX13
RcBBX1
RcBBX8
RcBBX19
RcBBX10
RcBBX17
RcBBX7
RcBBX9
RcBBX15
RcBBX22
RcBBX4
RcBBX20
RcBBX2
RcBBX11
RcBBX3
RcBBX18
RcBBX21
RcBBX6
RcBBX5
RcBBX16
Exon
Intron
UTR
1
2
3
4
5
6
7
8
9
10

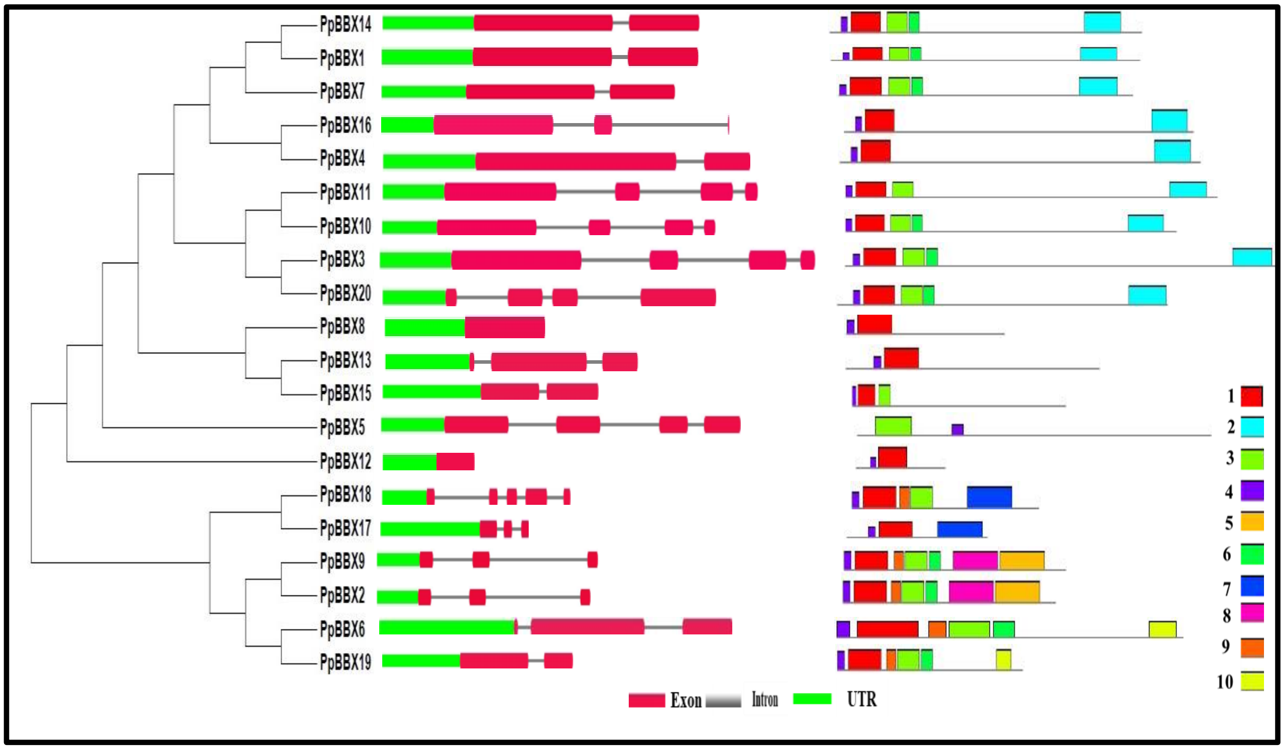
PpBBX14
PpBBX1
PpBBX7
PpBBX16
PpBBX4
PpBBX11
PpBBX10
PpBBX3
PpBBX20
PpBBX8
PpBBX13
PpBBX15
PpBBX5
PpBBX12
PpBBX18
PpBBX17
PpBBX9
PpBBX2
PpBBX6
PpBBX19
Exon
Intron
UTR
1
2
3
4
5
6
7
8
9
10

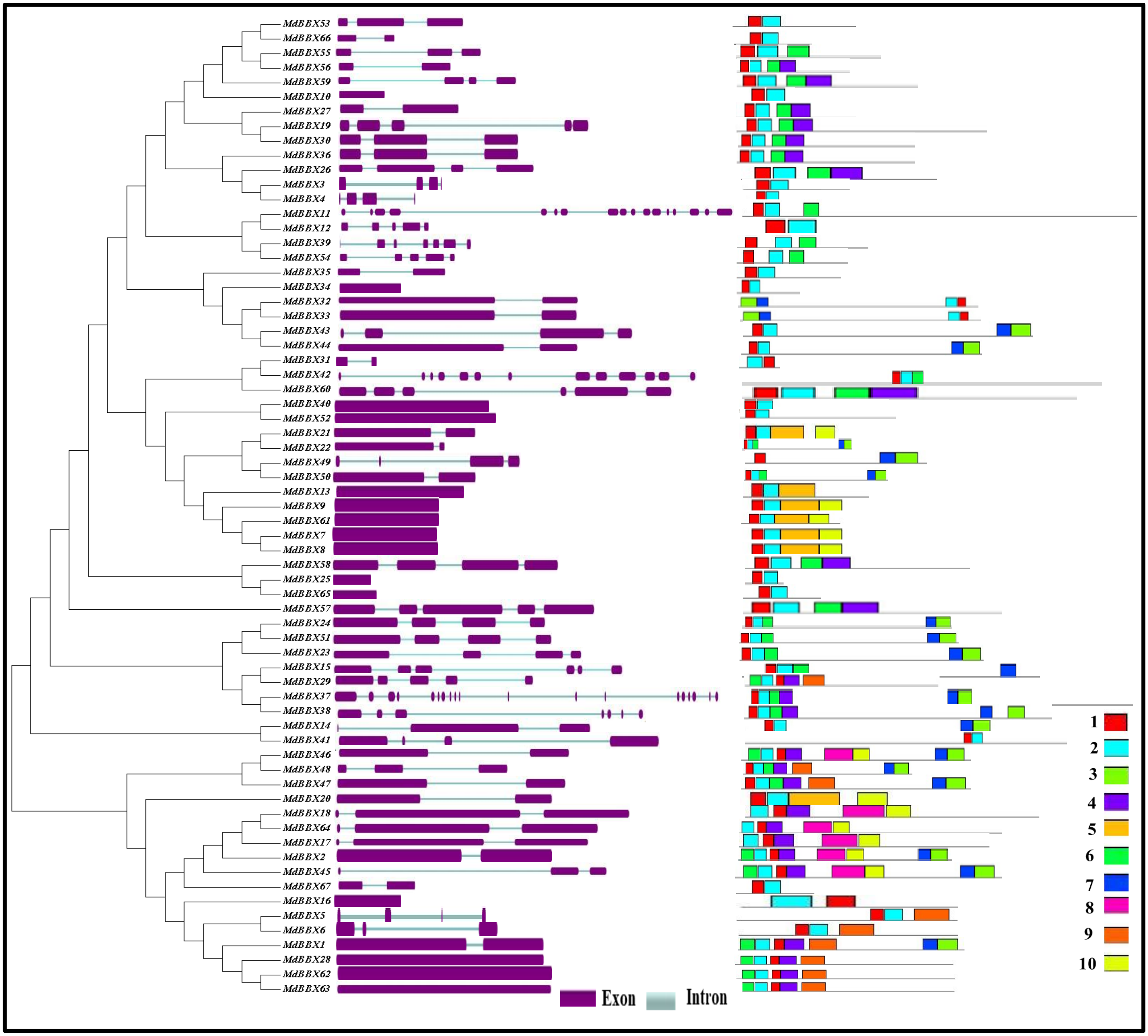

MdBBX53
MdBBX66
MdBBX55
MdBBX56
MdBBX59
MdBBX10
MdBBX27
MdBBX19
MdBBX30
MdBBX36
MdBBX26
MdBBX3
MdBBX4
MdBBX11
MdBBX12
MdBBX39
MdBBX54
MdBBX35
MdBBX34
MdBBX32
MdBBX33
MdBBX43
MdBBX44
MdBBX31
MdBBX42
MdBBX60
MdBBX40
MdBBX52
MdBBX21
MdBBX22
MdBBX49
MdBBX50
MdBBX13
MdBBX9
MdBBX61
MdBBX7
MdBBX8
MdBBX58
MdBBX25
MdBBX65
MdBBX57
MdBBX24
MdBBX51
MdBBX23
MdBBX15
MdBBX29
MdBBX37
MdBBX38
MdBBX14
MdBBX41
MdBBX46
MdBBX48
MdBBX47
MdBBX20
MdBBX18
MdBBX64
MdBBX17
MdBBX2
MdBBX45
MdBBX67
MdBBX16
MdBBX5
MdBBX6
MdBBX1
MdBBX28
MdBBX62
MdBBX63
Exon
Intron
1
2
3
4
5
6
7
8
9
10

Table S3: List of primers used for qRT-PCR

Name	Id	Forward Primer	Reverse primer
BBX1	MDP0000202669	CTTCTACGACTCGGTGAACTC	CGGATTCTCCATCGCCTTG
BBX7	MDP0000289278	ATGAAGAAGTGCGATCTGTGT	GATTGGCAGACATGGCAGAG
BBX14	MDP0000271388	TGGAGATTCAGCAGGATGTGTA	CCGCAGGAGCATTACTACTACT
BBX20	MDP0000297093	ATCCACTCTGCCAATCCTCTG	GCCTCATCCTCATCTTCCTCAT
BBX22	MDP0000198531	TGACGACGACGACGATGAG	CACCTCCATCACCATTGTTGAA
BBX26	MDP0000222881	AGGAGGAACTGGACGATTATGA	GAGTGGCAGATGTCTGGAAC
BBX27	MDP0000800387	TCTTCTGCTCTGCCGATGAA	CCTGCTGTGTTTCTGTGTGT
BBX30	MDP0000915501	AGCATCAACCTTCAACCAAGAA	CGGAAGCGTCTCTATCAAGTAC
BBX37	MDP0000302297	GGTTCTGGTGCTTCTAATGACA	TCTTGCCTTCCTTGACTCGTA
BBX38	MDP0000565292	CCGTTCCACCATCACCGTAT	ACAGAGAAGCATCAGGAGCAT
BBX39	MDP0000151848	CTAGTGATGTTCCTTGCTGTGA	GGTTGAAGCCCTAGTTCCTCT
BBX41	MDP0000273201	AGGTTGAAGAGGAAGTTGAAGG	TCATCACCATCTGCTTGTTCTC
BBX42	MDP0000298575	GCGATGCGAAGGTCCATTC	ACCTGTGTAACTGCTGATTGC
BBX43	MDP0000321380	TCTGCTTCCGAGCGACAT	CCTCCTCTAACTTCACTCTACC
BBX45	MDP0000713113	AGAAGAGGAAGAACCGCAAGT	GGAACGACGCCGAATTGTG
BBX47	MDP0000298635	TGACCAGCAGCAGCAACA	TGTGACTTAGCGAACCATTGTA
BBX49	MDP0000321180	GGTGAGCCAGGACGGTAAT	ACTATCTCCATCGCCATTGTTG
BBX51	MDP0000782323	CTCCCTGAAGACGATGACCTT	CAGCAACCGCATCTTGACAA
BBX52	MDP0000759984	GTCGTCATCGTCGGATTGTT	TCCATAGCATATTCCTCTTCGC
EF-1α	DQ341381	ATTCAAGTATGCCTGGGTGC	CAGTCAGCCTGTGATGTTCC
